# Angiotensin converting enzyme (ACE) inhibitors activity from purified compounds Fructus *Phaleria macrocarpa* (Scheff) Boerl

**DOI:** 10.1186/s12906-023-03889-x

**Published:** 2023-02-20

**Authors:** Aprilita Rina Yanti Eff, Hasniza Zaman Huri, Maksum Radji, Abdul Mun’im, F. D. Suyatna, Yonatan Eden

**Affiliations:** 1 Departement of Pharmacy, Faculty of Health Sciences Universitas Esa Unggul, Jakarta, Indonesia; 2 Department of Clinical Pharmacy & Pharmacy Practice, Faculty of Pharmacy Universiti Malaya, Kuala Lumpur, Malaysia; 3 Faculty of Pharmacy Universitas Indonesia, Jakarta, Indonesia; 4 Faculty of Medicine Universitas Indonesia, Jakarta, Indonesia

**Keywords:** *Phaleria macrocarpa*, ACE inhibitor, 6,4-dihydroxy-4-methoxybenzophenone–2-O-β-D-glucopyranoside, 4,4′-dihydroxy-6-methoxybenzophenone-2-O-β-D-glucopyranoside, Mangiferin

## Abstract

**Background:**

Mahkota Dewa [*Phaleria macrocarpa* (Scheff) Boerl.] fruit in vitro and *in- vivo* can decrease and prevent elevation of the blood pressure, lower plasma glucose levels, possess an antioxidant effect, and recover liver and kidney damage in rats. This study aimed to determine the structure and inhibitory activity of angiotensin-converting enzyme inhibitors (ACE) from the Mahkota Dewa fruit.

**Methods:**

The fruit powder was macerated using methanol and then partitioned by hexane, ethyl acetate, n-butanol, and water. The fractions were chromatographed on the column chromatography and incorporated with TLC and recrystallization to give pure compounds. The structures of isolated compounds were determined by UV-Visible, FT-IR, MS, proton (^1^H-NMR), carbon (^13^C-NMR), and 2D-NMR techniques encompassing HMQC and HMBC spectra. The compounds were evaluated for their ACE inhibitory activity, and the strongest compound was determined by the kinetics enzyme inhibition.

**Results:**

Based on the spectral data, the isolated compounds were determined as 6,4-dihydroxy-4-methoxybenzophenone–2-O-β-D-glucopyranoside (1), 4,4′-dihydroxy-6-methoxybenzophenone-2-O-β-D-glucopyranoside (2) and mangiferin (3). IC_50_ values of the isolated compounds 1, 2 and 3 were 0.055, 0.07, and 0.025 mM, respectively.

**Conclusion:**

The three compounds have ACE inhibitor and mangiferin demonstrated the best ACE inhibitory activity with competitive inhibition on ACE with the type of inhibition kinetics is competitive inhibition.

**Supplementary Information:**

The online version contains supplementary material available at 10.1186/s12906-023-03889-x.

## Background

Hypertension is a severe health problem that requires appropriate treatment, considering its relatively high prevalence, and complications may escalate morbidity and mortality and diminish life expectancy. The selection of antihypertensive drugs has encountered numerous changes because it is necessary to consider the efficacy, adverse effects, long-term use, and economic value. Indonesia and several other countries have extensively applied natural and herbal ingredients to treat and control the disease [1]. WHO explained that countries in Africa, Asia, and Latin America have been employing traditional medicine to complement the primary treatment. Even in Africa, as much as 80% of the population applies traditional medicine for primary treatment. Synthetic drugs can produce the effect more rapidly than traditional drugs; however, they tend to possess adverse effects and can cause more toxicity to the body. Traditional medicine owns advantages because it is uncomplicated to obtain, the raw materials can be grown in the surrounding environment, are cheap, and can made by everyone. Nevertheless, traditional medicine has some weaknesses, including the lack of standardized raw materials and safety and effectiveness tests that are not conducted regularly [2, 3].

Managing cardiovascular risk in hypertensive patients such as lipid disorders, diabetes, obesity, and smoking, is essential in maintaining blood pressure [3]. The objective of the treatment in hypertensive patients is to decrease SBP to < 140 mmHg and DBP to < 90 mmHg. The target for lowering blood pressure in patients with diabetes, chronic kidney disease, and coronary artery disease is < 130/80 mmHg [4].

ACE inhibitors are antihypertensive drugs that possess multiple modes of action. This drug prevents the formation of angiotensin II by inhibiting the angiotensin-converting enzyme. Angiotensin-converting enzyme (ACE) is an essential enzyme in synthesizing renin-angiotensin. This enzyme converts Angiotensin I to angiotensin II, a potent vasoconstrictor and activator of aldosterone secretion. Suppressing this enzyme causes vasodilation and decreases vascular resistance, which lowers blood pressure, aldosterone secretion, blood volume, and afterload [5]. Angiotensin II inhibitors decrease blood pressure by reducing peripheral vascular resistance, but they do not affect cardiac output or heart rate. These medications do not produce reflex sympathetic activation and are safe to use in persons with ischemic heart disease [6].

About 75 to 80% of the world’s population, particularly in developing countries, utilize herbal medicines to prevent and treat diseases, encompassing the treatment of hypertension. Herbal remedies are well-accepted by the body and possess lesser adverse effects. In the last three decades, many studies have been enacted to examine local plants which possess the potential as antihypertensives [7]. Several factors influence the mechanism of action of herbal medicines as antihypertensives, namely the role of smooth muscle cells, endothelial cells, ROS, and the role of the hormones endothelin-1 and angiotensin II (Ang II). Numerous phytochemicals in plants and herbs have helped manage hypertension and cardiovascular disease. Several factors contribute to using herbs alone or prescription medications to treat hypertension and other cardiovascular illnesses. Among them is their adherence to the “holistic concept” of medicine, which maintains that herbs are more cost-effective and safe than traditional medications and can be used to treat various health conditions [8].

Mahkota Dewa [*Phaleria macrocarpa* (Scheff) Boerl.] fruit is one of the plants extensively employed by the people of Indonesia because the price is low, easy to obtain, and generates various health benefits. The community has generally utilized this plant as traditional medicine, incorporating its use for hypertension [9]. The results of in-vitro studies on the methanol extract, fraction of petroleum ether, and ethyl acetate revealed that the extract and the fraction possessed activity as an ACE inhibitor with IC_50_ values of 161.7 μg/ml, 139.11 μg/ml, and 122.38 μg/ml, respectively [10]. Research on Mahkota Dewa fruit [*Phaleria macrocarpa* (Scheff) Boerl.] has been extensively conducted. However, most research is merely concerned with bioactivity, such as antimicrobial [11], vasodilator [12], cytotoxic [13], antidiabetic and antioxidant effects [14]. This study focuses on the ACE inhibitory activity of the active component from the Mahkota Dewa fruits as a chemical compound of *P. macrocarpa*. Various chemical constituents were isolated in *P. macrocarpa* fruit: glyceryl pentacosanoate, 1,7-dihydroxy-3,6-dimethoxyxanthone, and 1,6,7-trihydroxy-3-methoxyxanthone, dodecanoic acid, palmitic acid, and kaempferol-3-O-β-D-glucoside, ethyl stearate, and sucrose [15]. The objective of this study was to isolate and determine the active compound content of the Mahkota Dewa fruit and evaluate the ACE inhibitory activity of the isolated compounds. We report the successful isolation of three compounds with ACE inhibitory activity: 6,4′-dihydroxy-4-methoxybenzophenone–2-O-β-D-glucopyranoside, 4,4′-dihydroxy-6-methoxybenzophenone-2-O-β-D-glucopyranoside, and mangiferin. To our knowledge, the in-vitro ACE inhibition activity of three compounds has not previously reported.

## Material and method

### Materials

#### Sample collection

Mahkota Dewa fruit [*Phaleria macrocarpa* (Scheff) Boerl.] that has been ripe is maroon in color and obtained from cultivated plants from the Research Institute for Spices and Medicinal Plants (Balitro), Bogor, Indonesia. The fruits have been identified at Herbarium Bogoriensis, Center for Biological Research, Indonesian Institute of Sciences with the number identified 370/IPH/.1.02./if.8/III/2022. Plant materials were collected by the relevant guidelines and regulations of the Center for Plantation Research and Development, Agency for Agricultural Research and Development, and Ministry of Agriculture of the Republic of Indonesia. It followed the ethical guideline for plants. The voucher specimen was deposited in the Herbarium of Pharmacognosy Phytochemistry Laboratory, Faculty of Pharmacy, Universitas Indonesia.

### Method

These experiments were set up to isolate and characterize compounds from Mahkota Dewa fruit [*Phaleria macrocarpa* (Scheff) Boerl.] their fraction and pure compounds were evaluated against ACE inhibitor activity and enzyme inhibition kinetics.

### Extraction, isolation, and structure elucidation

The extraction was performed according to Zhang et al. 2020 with modification. A dried sample of Mahkota Dewa [*Phaleria macrocarpa* (Scheff) Boerl.] fruit (6 kg) was macerated using 15 L of 80% methanol for 3 × 24 hours [16]. The liquid extract was collected and concentrated in a rotary vacuum evaporator at 40 °C. The concentrated methanol extract (1032 g) was dispersed in 1000 mL of warm distilled water and partitioned by applying solvents, hexane, ethyl acetate, and butanol. The fractions were concentrated and dried using a vacuum oven at 40 °C. The results of the liquid-liquid fractionation of the viscous methanol extract employed hexane, ethyl acetate, and n-butanol as solvents and water yielded 3.01, 17.11, 17.44, and 62.44%, respectively. Furthermore, the ACE inhibitory activity assay was performed from extract and fraction using captopril as a positive control. The fraction of ethyl acetate that resulted in the smallest IC_50_ value was chromatographed by liquid column chromatography (Ø = 3.8 and t = 49.5 cm) with 180 g of silica gel 60 as a stationary phase. It was eluted by a solvent gradient, starting from n-hexane: methanol (100:0, 80:20, 60:40, 40:60, 20:80, 0:100) and ethyl acetate: methanol (100:0, 80:20, 60:40, 40:60, 20:80, 0:100), respectively. 100 ml fractions were collected and monitored by analytical TLC with chloroform-methanol-water (70:20:2) (v/v) as a mobile phase. Compounds separated were detected under UV light at λ = 366 nm. The obtained subfractions were merged based on TLC similarity. Then the sub-fraction was recrystallized using 50% chloroform (in methanol) to obtain powder compounds. The three isolate including compound 1 (317,1 mg), compound 2 (383,2 mg) and compound 3 (217,7 mg), respectively. The structure of three purified compounds was identified by analyzing their spectra obtained from UV-Visible (Shimadzu UV 1601), FT-IR spectrophotometer (Shimadzu), mass spectroscopy (Water, Milford, MA, USA), proton (^1^H-NMR) and carbon (^13^C-NMR) spectroscopy (JEOL JNM, Japan), and 2D-NMR techniques encompassing HMQC and HMBC (JEOL JNM, Japan) at Pusat Penelitian Kimia, Lembaga Ilmu Pengetahuan Indonesia Serpong, Tangerang, Banten, Indonesia. The ACE inhibitor activity from purified compounds was determined using the same method as the extract and fraction. The active compound with the best IC_50_ was examined for enzyme inhibition kinetics.

### In-vitro ACE inhibitor activity assay and enzyme inhibition kinetics

This assay was conducted on extract, fraction and three purified compounds using captopril as a positive control at the concentration of 0,01, 0,05, 0,5, 1,25 dan 6,25 mM. In a test tube, 50 μL of the solution assay was added with 50 μL of 8 mM hippuryl-histidyl-leucine (HHL) substrate solution and pre-incubated at 37 °C for 10 minutes, followed by the addition of 100 μL of the ACE, homogenization by vortex mixer, and incubation for 90 minutes at 37 °C. Further, 250 μL of 1 N HCl was added to the mixture to stop the enzymatic reaction. The hippuric acid was produced, while the enzymatic reaction was extracted by 1.5 mL ethyl acetate and later separated from the mixture by centrifugation. The mixture was then concentrated by heating at 100 °C and dispersed in 3 mL of demineralized aqueous to have its absorbance measured by spectrophotometer at 228 nm [17]. The hippuric acid concentration was utilized to calculate the percentage of inhibition (%), and the IC_50_ value was then calculated using the percentage inhibition obtained. The IC_50_ value was determined as the peptide concentration in mg/mL was demanded to reduce 50% of ACE activity by regression analysis of the ACE inhibition versus ACE concentration percentage. The examination of the kinetics of enzyme inhibition was carried out at several concentrations of HHL substrates, which are 4, 6, 8, 10, and 12 μg/ml. The samples administered were compound with the lowest IC_50_ value. An inhibition kinetics test was employed to determine the inhibition mechanism of these compounds. It was performed type of Inhibition was determined by the Lineweaver-Burk method to acquire the Michaelis-Menten kinetic constant. The Michaelis-Menten kinetic constant (Km) is assessed based on the regression equation y = a + bx, in which x is the substrate concentration [S], and y is the absorbance of the sample [18].

## Results

### In-vitro ACE inhibitor activity from fraction

The ACE inhibitory activity results in a fraction of hexane, ethyl acetate, butanol, and water at a concentration of 125 μg/ml and IC_50_ value can be seen in Table 1.

**Table 1:** ACE inhibitory activity from the fraction of hexane, ethyl acetate, butanol and water

Sample	Inhibitory activity ± SD (%)	IC_**50**_ ± SD (μg/mL)
Methanol extract		122.38 ±
Hexane fraction	45.94 ± 0 .52	236.06 ± 0.1
ethyl acetate fraction	62.52 ± 0.7	36.78 ± 0.11
Butanol fraction	39.73 ± 0.52	206.15 ± 0.57
Water fraction	41.63 ± 0.28	152.3 ± 0.4

The ethyl acetate fraction has the smallest IC_50_ value, and then it is chromatographed with liquid column chromatography for isolation, purification, and identification of the active compound.

### Spectroscopy analysis of isolated compound

**Fig. 1:**
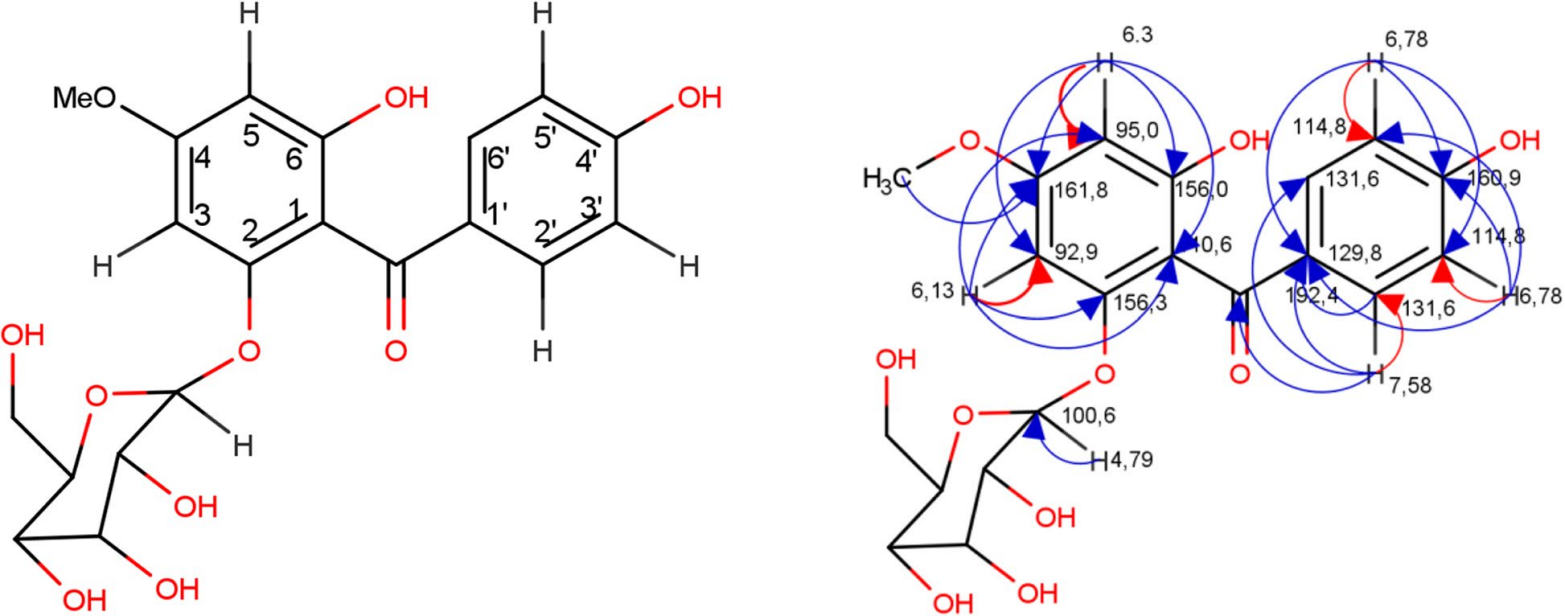
Two–dimensional NMR spectrum of compound 1*.* HSQC: heteronuclear single quantum coherence (red arrow); HMBC: heteronuclear multiple bond correlation (blue arrow)

This study successfully isolated and identified three majority compounds from the Mahkota Dewa fruit [*Phaleria macrocarpa* (Scheff) Boerl]. Compound 1 is a white powder with yellow fluorescence at a wavelength of 366 nm. The IR (cm^− 1^): 3215.4, 3367.82, 2692.72, 2603.99, 1932.74, 1844.01, 1791.93 cm-1, 1716.7, 1549.1, 1508.4, 1456.3, 1170 and 1006.26. UV (λmax) 291 nm. Chemical shift (δC) at 100.7; 77.1; 76.6; 73.1; 69.7 and 60.7 ppm and chemical shifts C 100.7 ppm and H 4.8 ppm with constant coupling (J) = 8 Hz. LC-MS data revealed that the compound possesses a molecular weight of [M^+^] = 422.43. Data for ^1^H-NMR and ^13^C-NMR (DMSO solvent) (Table 2). Two–dimensional NMR spectrum of compound 1 can be seen in Fig. 1.

**Table 2:** Spectral data of ^1^H, ^13^C of compound 1

C Position	Compound 1		Heteronuclear multiple bond correlation
	^**1**^HNMR (δ = ppm, ***J*** = Hz)	^**13**^CNMR (ppm) (δ = ppm, ***J*** = Hz)	
1	–	110.6	–
2	–	156.3	–
3	6.13(d, *J* = 3.0)	92.9	C1, C2, C4, C5
4	–	161.8	–
5	6.3 (d, *J* = 2.5)	95.0	C1, C3, C4, C6
6	–	156.0	–
7	–	192.4	–
OCH_3_	3.73 (s)	54.9	C4
1′	–	129.8	–
2′	7.58 (d, *J* = 9.1)	131.6	C1′, C6’, C7
3′	6.78 (d, *J* = 8.4)	114.8	C1’, C4’, C5’
4′	–	160.9	–
5′	6.8 (d, J = 8,6)	114.8	C1′, C4′, C3′
6′	7.58 (d, J = 9.1)	131.6	C1′, C2′, C7
1′′	4.79 (d, J = 7.8)	100.6	C2
2′′	2.92 (dd, J = 3.9, 3.9)	73.1	C1′′; C 3′′
3′′	3.18 (dd, J = 5.9, 5.7)	76.6	C2′′; C4′′
4′′	3.01 (dd, J = 7.8, 4.6)	69.7	C5′′; C6′′
5′′	3.29 (m)	77.1	C6′′
6′′	3,39 (m)	60,7	C5′′

**Fig. 2:**
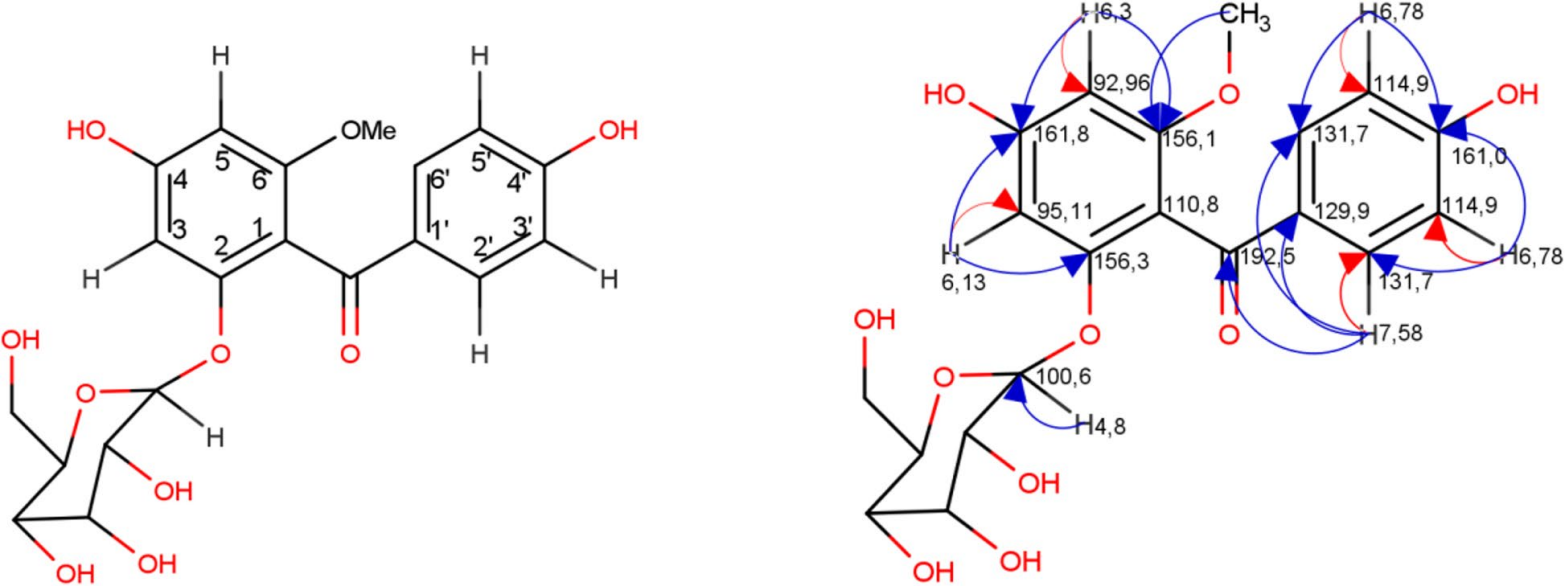
Two–dimensional NMR spectrum of compound 2. HSQC: heteronuclear single quantum coherence (red arrow); HMBC: heteronuclear multiple bond correlation (blue arrow)

The characterization of compound 2 is a brownish yellow solid, easily soluble in DMSO and methanol. Spraying this compound with FeCl_3_ stains generates a dark blue color and yellow with AlCl_3_. UV (λmax) 296 nm. IR (cm^− 1^) = 3365.9, 2947.3, 1653.05, 1604.83, 1508.8 and 1471.4. The LCMS m/z 445[M^+^ 23(Na)]. Data for ^1^H-NMR and ^13^C-NMR (DMSO solvent) displayed that compound 2 (Table 3). Two–dimensional NMR spectrum of compound 2 can be perceived in Fig. 2.

**Table 3:** Spectral data for ^1^H, ^13^C for compound 2

C Position	Compound 2		Heteronuclear multiple bond correlation
	^**1**^HNMR (δ = ppm, ***J*** = Hz)	^**13**^CNMR (ppm) (δ = ppm, ***J*** = Hz)	
1	–	110,7	–
2	–	156,1	–
3	6,13(d, *J* = 2,0)	95,1	C4, C2
4	–	161,8	–
5	6,3 (d, *J* = 2,0)	92,9	C6, C4
6	–	156,3	–
7	–	192,5	–
OCH_3_	3,73 (s)	55	C6
1′	–	129,8	–
2′	7,58 (d, *J* = 7,2)	131,7	C1’, C6’, C7
3′	6,78 (d, *J* = 8,6)	114,9	C2’, C4’
4′	–	161,0	–
5′	6,78 (d, *J* = 8,6)	114,9	C4’, C6’
6′	7,58 (d, *J* = 8,4)	131,7	C1’, C2’, C7
1′′	4,8 (d, *J* = 8)	100,6	–
2′′	2,93 (dd, *J* = 3,9, 3,9)	73,2	–
3′′	3,20 (dd, *J* = 5,9, 5,7)	76,6	–
4′′	3,02 (dd, *J* = 7,8, 4,6)	69,8	–
5′′	3,4 (m)	77,2	–
6′′	3,7 (m)	60,8	–

**Fig. 3:**
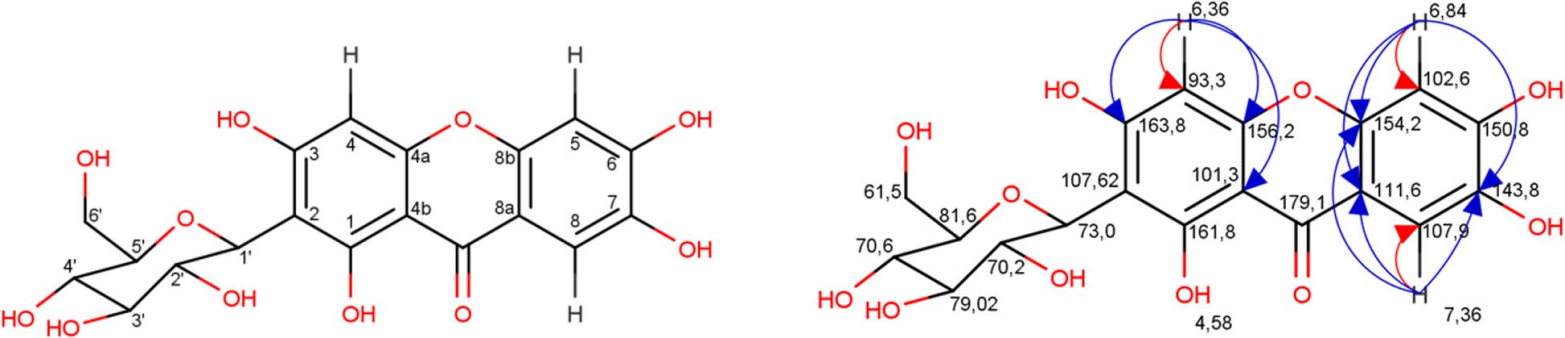
Two–dimensional NMR spectrum of compound 3. HSQC: heteronuclear single quantum coherence (red arrow); HMBC: heteronuclear multiple bond correlation (blue arrow)

Compound 3 is characterized as a light-yellow solid, easily soluble in DMSO and methanol. The results of TLC with FeCl_3_ stains generates a dark blue color, and AlCl_3_ produces a yellow color. Compound 3 is foreseen to be a phenolic group. UV (λmax) 292 nm. IR (cm^− 1^): 3367.8, 2941.2, 1622.9, 1525, and 1490. The LCMS 445 m/z [M^+^ 23(Na)]. Data for ^1^H-NMR and ^13^C-NMR (DMSO solvent) presented Table 4. Figure 3 depicts the Two–dimensional NMR spectrum in compound 3.

**Table 4:** Spectral data of ^1^H, ^13^C of compound 3

C Position	Compound 3		Heteronuclear multiple bond correlation
	^**1**^HNMR (δ = ppm, ***J*** = Hz)	^**13**^CNMR (ppm) (δ = ppm, ***J*** = Hz)	
1	–	161.8	
2	–	107.6	
3	–	163.8	
4	6,36 (s)	93.3	C3, C-4a, C-4b
5	6,84 (s)	102.6	C-8a, C-8b, C7
6	–	150.8	
7	–	143.8	
8	7,36 (s)	107.9	C-O, C-8a, C-8b, C-7
9	–	179.1	
4a	–	156.2	
4b	–	101.3	
8a	–	111.6	
8b	–	154.2	
CO	–	179.0	
1′	4,58 (d, *J* = 9,75)	73.0	C-1, C-3, C-2, C6’
2′	4,05 (t)	70.2	C-1’, C-3’
3′	3,20(m)	79.0	C-5′, C-4’
4′	3,19(m)	70.6	C-5’
5′	3,16 (m)	81.6	C4’, C-6’
6′	3,67 (d, *J* = 11,1)	61.5	C5’

### The ACE inhibitory activity results of three active compound

We conducted an inhibitory assay to identify which of the active substances from mahkota dewa was responsible for the inhibition of ACE. The ACE inhibitory activity results from the three purified compounds at a concentration of 1.25 μg/ml and IC_50_ value given in Table 5.

**Table 5:** ACE inhibitory activity from the three purified compounds

Sample	inhibitory activity ± SD (%))	IC_**50**_ ± SD (μg/mL)
Compound **1**	77.48 ± 0.5	0.055 ± 0.006
Compound **2**	85.17 ± 0.23	0.07 ± 0.002
Compound **3**	86.99 ± 0.02	0.025 ± 0.001
Captopril	90.61 ± 0.41	0.017 ± 0.003

### Enzyme inhibition kinetics

**Fig. 4:**
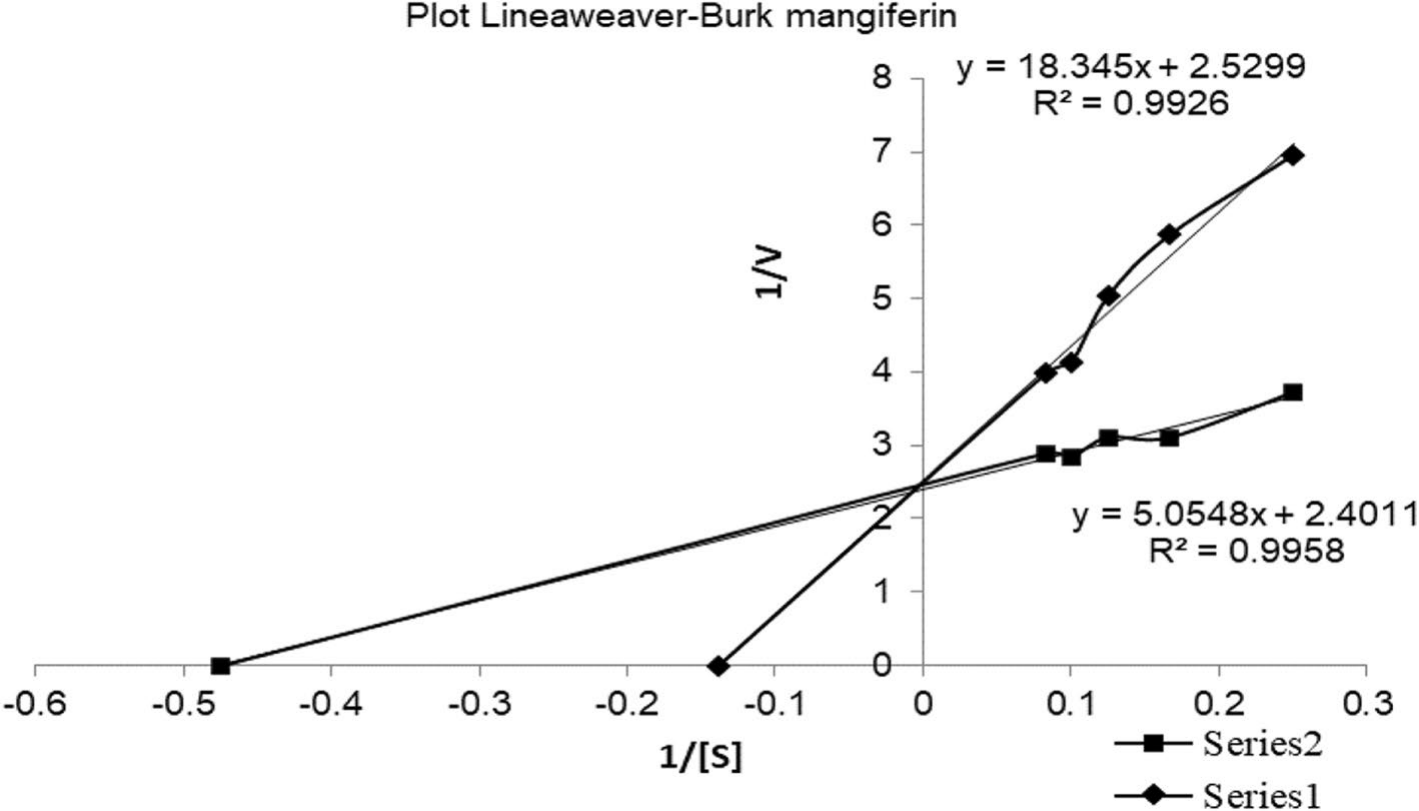
Lineaweaver-Burk plot of mangiferin. Description: series 1: Without Inhibitor, series 2: mangiferin

Kinetic analysis of ACE inhibition was conducted by administering the Lineweaver-Burk plot (Fig. 4). The samples administered were compound with an IC_50_ value of 0.025 mM (compound 3). The Lineweaver-Burk graph is generated from relationship of 1/[S] to 1/V.

## Discussion

The renin-angiotensin-aldosterone system regulates arterial blood pressure and electrolyte balance through the angiotensin-converting enzyme (ACE), a glycosylated zinc dipeptidyl-carboxypeptidase, which is one of its primary components [19]. This study successfully isolated, identified, and elucidated the structure of three active compounds as ACE inhibitors from Mahkota Dewa [*Phaleria macrocarpa* (Scheff) Boerl.] fruit and an assay of ACE inhibitor activity. Table 1 shows that the ethyl acetate fraction of fruit (*Phaleria macrocarpa* (Scheff) Boerl.) has the lowest IC_50_ value of 36.78 μg/mL. Our previous study on Petroleum ether extract, ethyl acetate extract, and methanol extract yielded IC_50_ values of 161.7 g/mL, 139.11 g/mL, and 122.38 g/mL, respectively [10].

Compound 1 is a white powder with yellow fluorescence at a wavelength of 366 nm. It is included in a xanthone group. The FTIR spectrum of compound 1 at wave number ν = 3215.4 cm^− 1^ and 3367.82 cm^− 1^ indicated the occurrence of OH bond stretching vibration at wavenumber ν = 2692.72 cm^− 1^ and 2603.99 cm^− 1^ which infers the presence of stretching vibration asymmetric = CH. The occurrence of C=O bonds is identified by the wavenumber ν = 1932.74 cm^− 1^, 1844.01 cm^− 1^, 1791.93 cm^− 1^, and 1716.7 cm^− 1^. In contrast, the occurrence of aromatic C=C is unveiled by the wavenumber ν = 1549.1 cm^− 1^, 1508.4 cm^− 1^, 1456.3 cm^− 1^ identifying the presence of an aromatic ring HC=CH, and the occurrence of CO ester bonds 1170 cm^− 1^ and 1006.26 cm^− 1^. The UV spectroscopy results presented a maximum absorption (λmax) at a wavelength of 291 nm, which indicates the substitution of C=O group on the aromatic ring. From the proton and carbon NMR spectra, it can be perceived that CH_3_ is –OCH_3_. Chemical shifts (δC) at 100.7; 77.1; 76.6; 73.1; 69.7 and 60.7 ppm are specific signals for glucoside groups, and chemical shifts C 100.7 ppm and H 4.8 ppm with constant coupling (J) = 8 Hz, which indicates that the glucoside moieties are O-glycosylated in the position. LC-MS data revealed that the compound possesses a molecular weight of 422 with the molecular formula C_20_H_22_O_10_. Data for ^1^H-NMR and ^13^C-NMR spectra (DMSO solvent) presented that compound 1 owned a similar structure to 6,4′-dihydroxy-4-methoxybenzophenone-2-*O*-β-D-glucopyranoside. This compound has IC_50_ value 0.055 ± 0.006 μg/ml. Winarno H, 2019 isolated 6,4′-dihydroxy-6-methoxybenzophenone-2-O-β-D-glucopyranoside from the bark of Mahkota Dewa and assessed its activity against leukemia l1210 cell line with an IC_50_ value of 5.1 μg/ml [20].

The characterization of compound 2 is a brownish-yellow solid, easily soluble in DMSO and methanol. Spraying this compound with FeCl_3_ stains generates a dark blue colour and yellow with AlCl_3_. It is presumed that compound 2 belongs to the phenolic group. The UV spectrum displayed maximum absorption at a wavelength of 296 nm, which implies a substituted -C=O group on the aromatic ring. The FTIR spectrum presented –OH groups at wave number ν = 3365.9 cm^− 1^, and saturated –CH groups at wave number ν = 2947.3 cm^− 1^. The absorption characteristics at wave numbers ν = 1653.05 cm^− 1^ and 1604.83 cm^− 1^ indicate the presence of a –C=O group. At wave numbers 1508.8 cm^− 1^ and 1471.4 cm^− 1^, it is revealed the presence of an aromatic ring –C=C-. The LCMS results uncovered that compound 2 possesses a molecular weight of 422 with the molecular formula C_20_H_22_O_10_. Data for ^1^H-NMR and ^13^C-NMR spectra (DMSO solvent) displayed that compound 2 was 4,4′-dihydroxy-6-methoxybenzophenone-2-O-β-D-glucopyranoside [20, 21]. The IC_50_ value of this compound is 0.07 ± 0.002 μg/ml. Zhang et al. 2006 have successfully isolated three glucosides from mahkota dewa; there are (4,4′-dihydroxy-6-methoxybenzophenone-2-O-β-D-glucopyranoside (mahkoside A), mangiferin and kaempferol-3-O-β-D-glucoside however, these three components were not tested for their activities [21]. Zhang et al., 2012 isolated 2,4′,6-trihydroxy-4-methoxy-benzophenone-2-O-β-D-glucoside and stated that this structure is a mahkoside A [22], but this study did not show the NMR data. While the studies conducted by Winarno et al., 2009 [20], and Zhang et al. 2006 [21] stated that Mahkoside A is 4,4′-dihydroxy-6-methoxybenzophenone-2-O-β-D-glucopyranoside. Based on the ^1^H, ^13^C, and HMBC data similarities, we represent that 4,4′-dihydroxy-6-methoxy benzophenone-2-O-β-D-glucopyranoside is mahkoside A.

Compound 3 is characterized as a light-yellow solid, easily soluble in DMSO and methanol. The results of TLC with FeCl_3_ stains generate a dark blue colour, and AlCl_3_ produces a yellow colour. Compound 3 is foreseen to be a phenolic group. The UV spectrum revealed maximum absorption at a wavelength of 292 nm, which indicates a substituted -C=O group on the aromatic ring. The FTIR spectrum presented the occurrence of –OH group at wave number ν = 3367.8 cm^− 1^ and the saturated –CH group at wave number = 2941.2 cm^− 1^. The absorption characteristic at wave number ν = 1622.9 cm^− 1^ implies the presence of –C=O group, and at wave numbers 1525 cm^− 1^ and 1490 cm^− 1^ presents the occurrence of an aromatic ring –C=C-. The LCMS results revealed that compound 3 possesses a molecular weight of 422 with the molecular formula C_19_H_18_O_11_. Data for ^1^H-NMR and ^13^C-NMR spectra (DMSO solvent) displayed that compound 3 has a structure similar to mangiferin [23], with an IC_50_ value is 0.025 ± 0.001 μg/ml.

Ramdani et al. 2017 isolated and identified nine active compounds from Mahkota Dewa fruit using 90% ethanol solvent and then partitioned them with n-hexane/H_2_O and ethyl acetate/H_2_O. Glyceryl pentacosanoate, a recently discovered chemical, was recognized as one of them. Additionally, the isolation of two xanthones, 1,7-dihydroxy-3,6-dimethoxyxanthone and 1,6,7-trihydroxy-3-methoxyxanthone, from *P. macrocarpa* is first described [15].

Natural ACE inhibitors have reportedly been shown to offer lengthy antihypertensive benefits without appreciable toxicity or adverse side effects on the human body. Additionally, most chemicals originating from plants positively affect other aspects of normal metabolism [24]. Table 5 shows ACE inhibitory activity from the three purified compounds. Compound 3 has the lowest IC_50_ of 0.025 ± 0.001 μg/ml, while the IC_50_ of captopril is 0.017 ± 0.003 μg/ml. Captopril is more frequently utilized as an ACE inhibitor because it has a free radical scavenger activity and is used as an antihypertensive and heart failure medicine [25]. The activity of ACE inhibition from medicines or plant extracts can be detected using various ACE inhibitory activity assay methods. Using a substrate of hippuryl-histidyl-leucine (HHL), we used the Cushman and Cheung approach to the data from this study. The ACE will hydrolyze HHL into hippuric acid (HA). A UV-visible spectrophotometer was used to measure the HA at a wavelength of 228 nm to describe the ACE activity. An ACE inhibitor will result in a lower concentration of HA being produced [26]. Plant-based ACE inhibitors have therapeutic potential in treating hypertension and other anomalies associated with diabetes. Most ACE inhibitory plant metabolites are peptides, protein hydrolysates, phenolics, flavonoids, terpenoids, and alkaloids [24]. Meanwhile, the potential for ACE inhibitors from the benzophenone group has not been reported. In this study, we report three benzophenone compounds that have activity as ACE inhibitors, namely 6,4-dihydroxy-4-methoxybenzophenone-2-O-β-D-glucopyranoside, 4,4′-dihydroxy-6-methoxybenzophenone-2-O-β-D-glucopyranoside and mangiferin.

The active compound with the smallest IC_50_ value was examined for enzyme inhibition kinetics. Figure 4 shows that the V_max_ values for isolates (mangiferin) and without inhibitors are almost the same. However, the Km values are different based on the calculation results of the Michaelis-Menten constant. Thus, it may be said that compound 3 (mangiferin) inhibits ACE activity by competitively inhibiting kinetics. The term “substrate analogs” refers to substances that have a structure comparable to the substrate and act as competitive inhibitors in the inhibition type [27, 28]. Competitive inhibitors compete with the substrate for the enzyme’s active site so that the enzyme cannot produce an enzyme-substrate complex. However, when the concentration of the competitive inhibitor is greater than that of the substrate, it binds to the active binding site to form an enzyme inhibitor complex (EI), which ultimately prevents the formation of any product. In a typical enzymatic reaction, Vmax is the reaction’s maximal rate. Km, also known as the Michaelis-Menten constant, is the substrate concentration halfway between Vmax and Km [29]. Km is an appropriate measurement unit for determining the reaction rate as substrate concentration increases. The graph reaches a plateau because there are no more enzymes other than the substrates already bound, and all the enzyme molecules are saturated with the available substrates. That indicates any other available substrates not used are excluded due to the lack of enzymes, which may be a rate-limiting factor for the reaction rate. However, in competitive inhibition, Vmax remains unaltered, or the reaction reaches its normal Vmax, but a more significant concentration of Km is needed to get there [28].

ACE is an enzyme accommodating peptidyl dipeptide hydrolase. The active site of ACE encompasses three parts, which are a carboxylate binding site such as the guanidium group of arginine, a pocket encompassing the hydrophobic side chain of the terminal amino acid residue, and the Zn ion. Flavonoids and other polyphenols function by forming chelate complexes with the Zn atom at the Zn metallopeptidase active ion center or generating hydrogen bridges between the inhibitor and amino acids close to the active center the enzyme [30]. Flavonoids, prominently flavan-3-ol and procyanidin, inhibit ACE competitively with the substrate in the enzyme’s active site, according to kinetic studies. Anthocyanins, delphinidin-3-O sambubioside, and cyanidin-3-O-sambubioside, all isolated from *Hibiscus sabdariffa*, were demonstrated to inhibit ACE at the active site in a competitively [31].

## Conclusions

Three compounds as new ACE inhibitors, namely 6,4′-dihydroxy-4-methoxybenzophenone–2-O-β-D-glucopyranoside, 4,4′-dihydroxy-6-methoxybenzophenone-2-O-β-D-glucopyranoside, and mangiferin were isolated and purified from Mahkota Dewa fruit [*Phaleria macrocarpa* (Scheff) Boerl]. This study supports Mahkota Dewa fruit as an antihypertensive used in conventional medicine. Mangiferin has the lowest IC50 value of 0.025 ± 0.001 μg/mL compared to 6,4′-dihydroxy-4-methoxybenzophenone–2-O-β-D-glucopyranoside, 4,4′-dihydroxy-6-methoxybenzophenone-2-O - β-D-glucopyranoside and inhibits ACE activity by competitively inhibiting kinetics.

## Supplementary Information

**Additional file 1.**

## Data Availability

The data sets generated and/or analyzed during the current study are available from the corresponding author on reasonable request.
