# Supplementary material for: Angiotensin converting enzyme (ACE) inhibitors activity from purified compounds Fructus Phaleria macrocarpa (Scheff) Boerl
Source: BMC Complement Med Ther. 2023 Feb 20;23:56. doi: 10.1186/s12906-023-03889-x (PMC9940430; doi:10.1186/s12906-023-03889-x)
Supplement: Supplementary file 1 — Additional file 1. [file 12906_2023_3889_MOESM1_ESM.pdf]

Compound 1

IR spectra

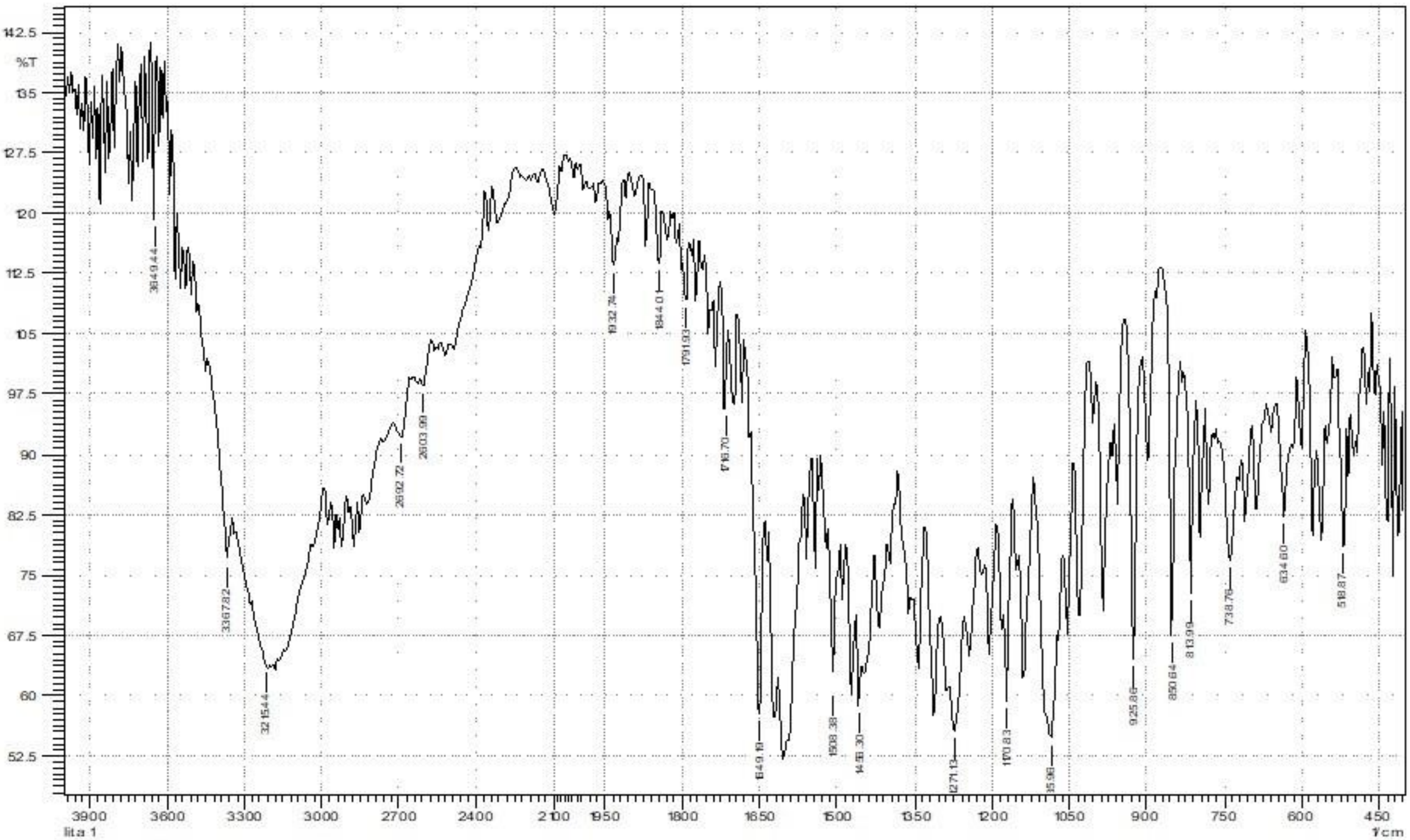

LCMS spectra

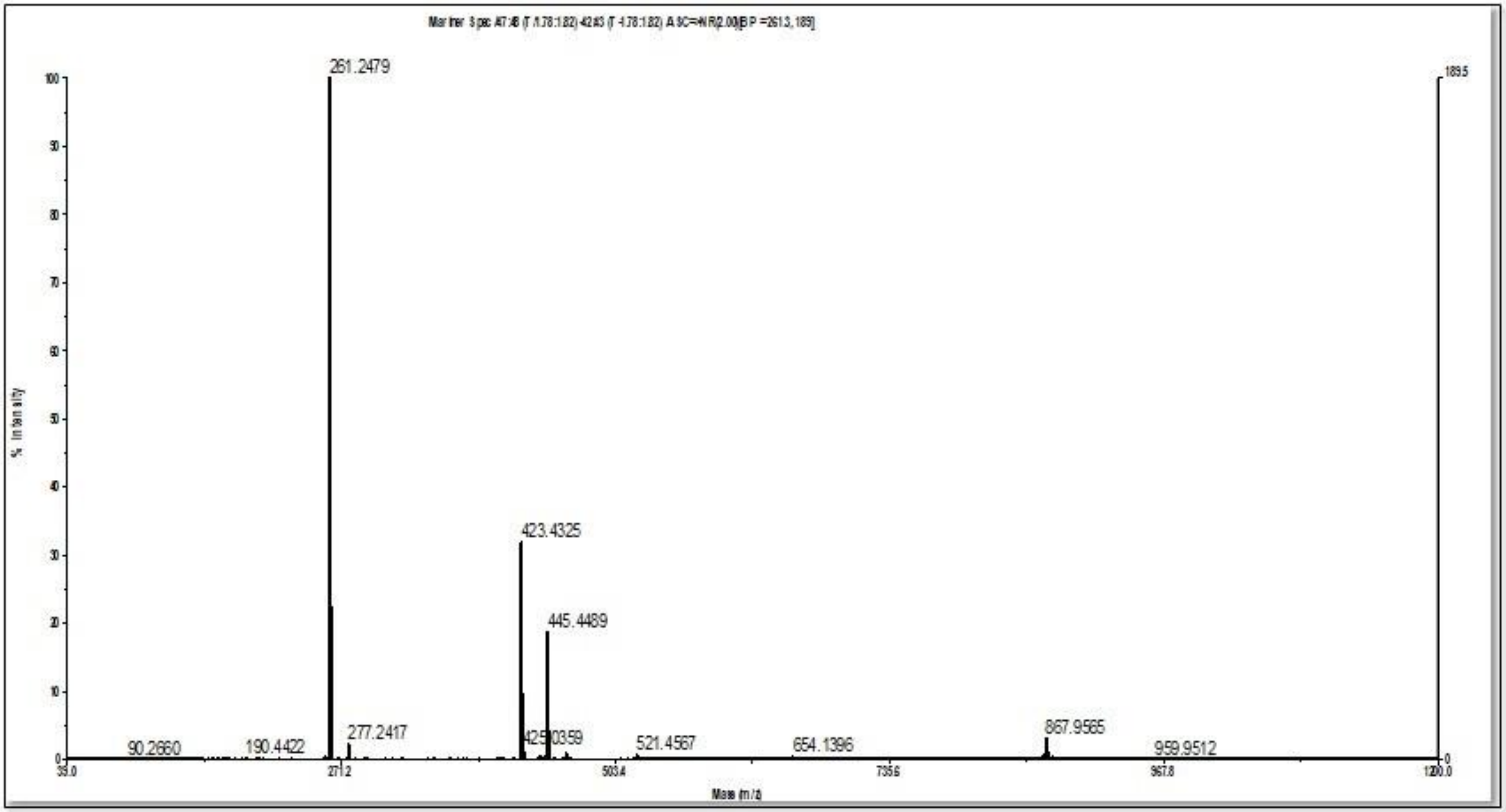

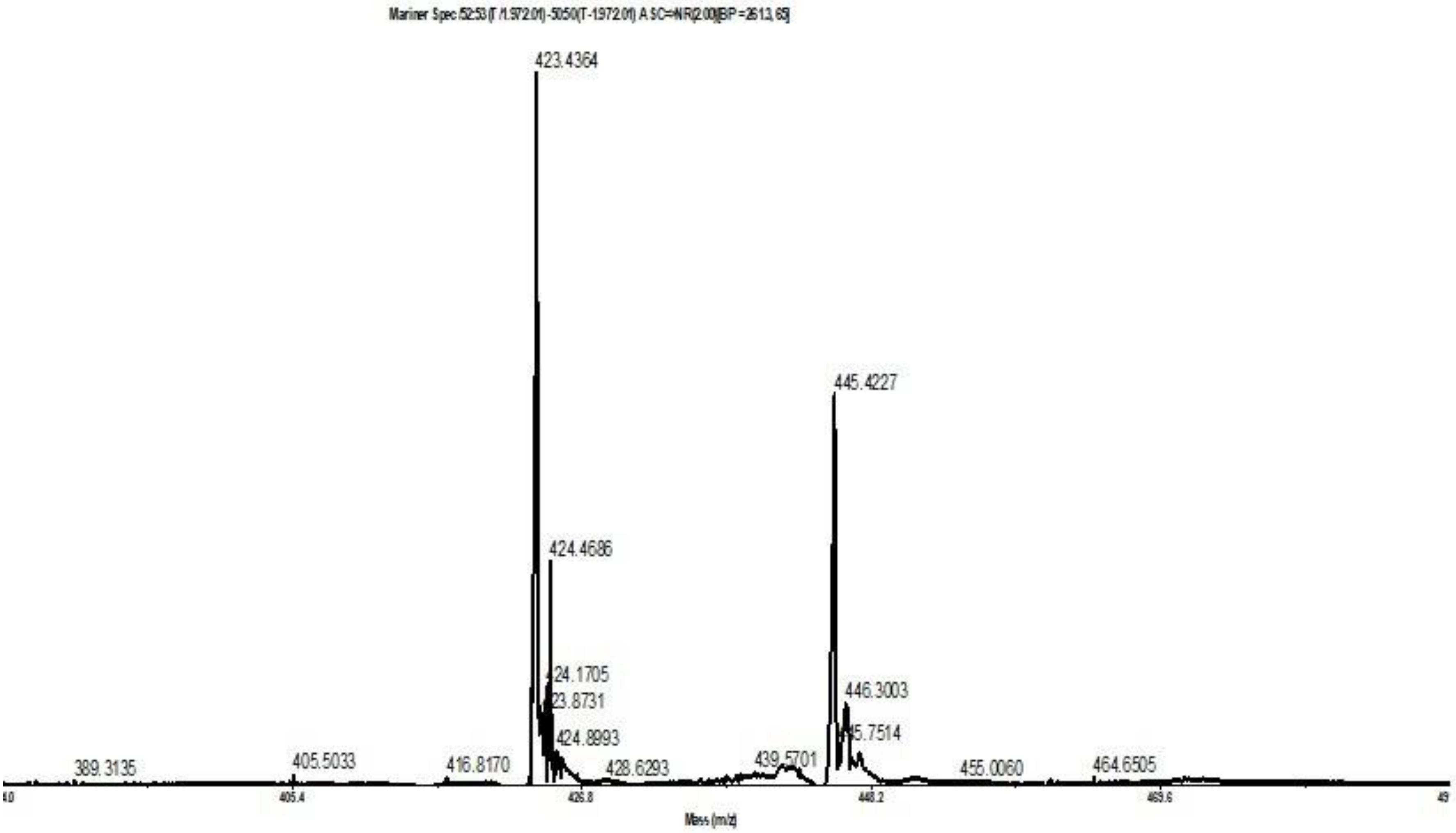

Mariner Spec /52:53 (T /1.972.01) -50:50(T -1.972.01) A SC=NR(2.00)[BP = 2613, 65]
423.4364
424.4686
424.1705
23.8731
424.8993
445.4227
446.3003
45.7514
389.3135
405.5033
416.8170
428.6293
439.5701
455.0060
464.6505
405.4
426.8
448.2
469.6
Mass (m/z)

NMR spectra, HMBC and HSQC

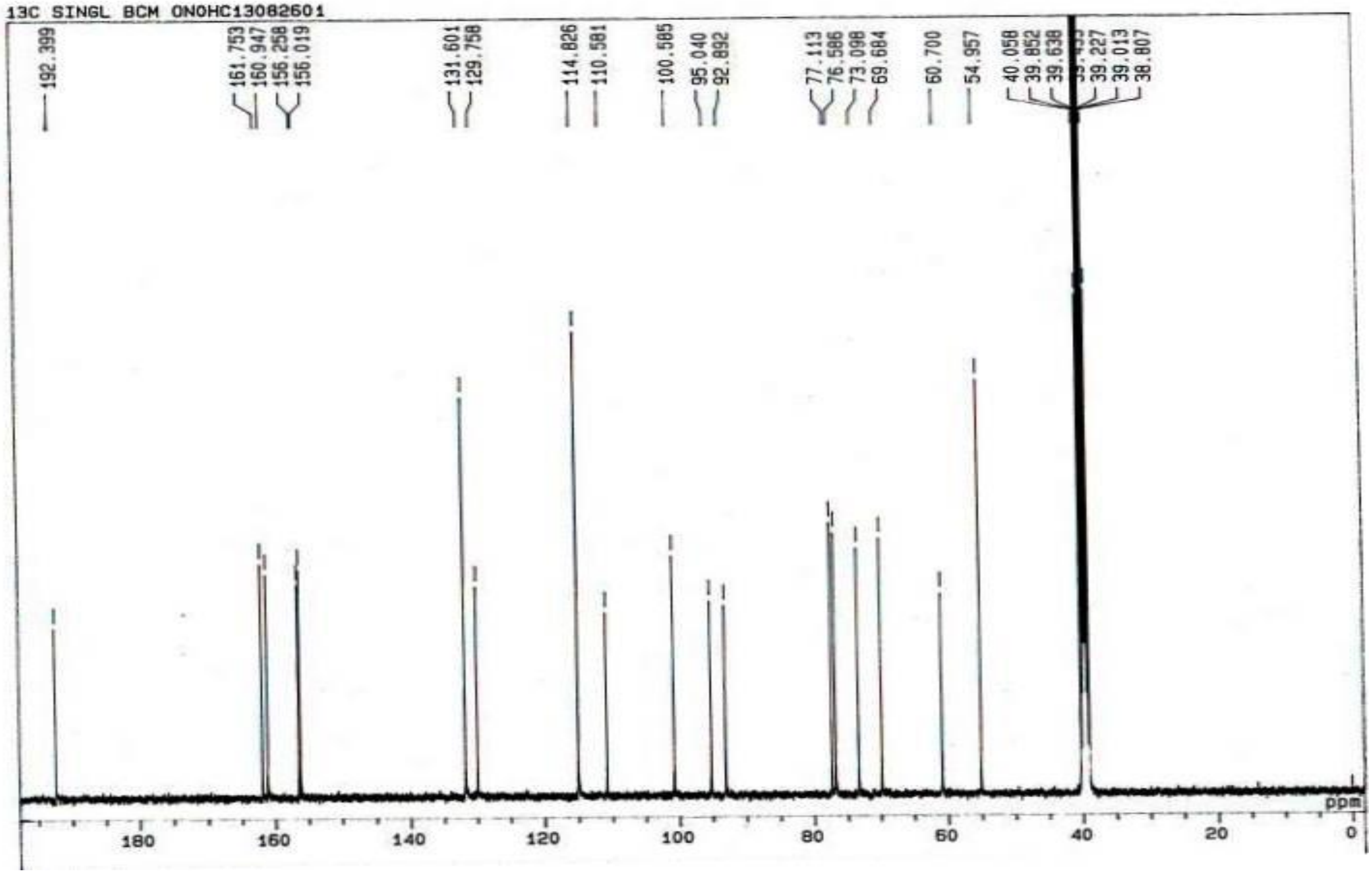

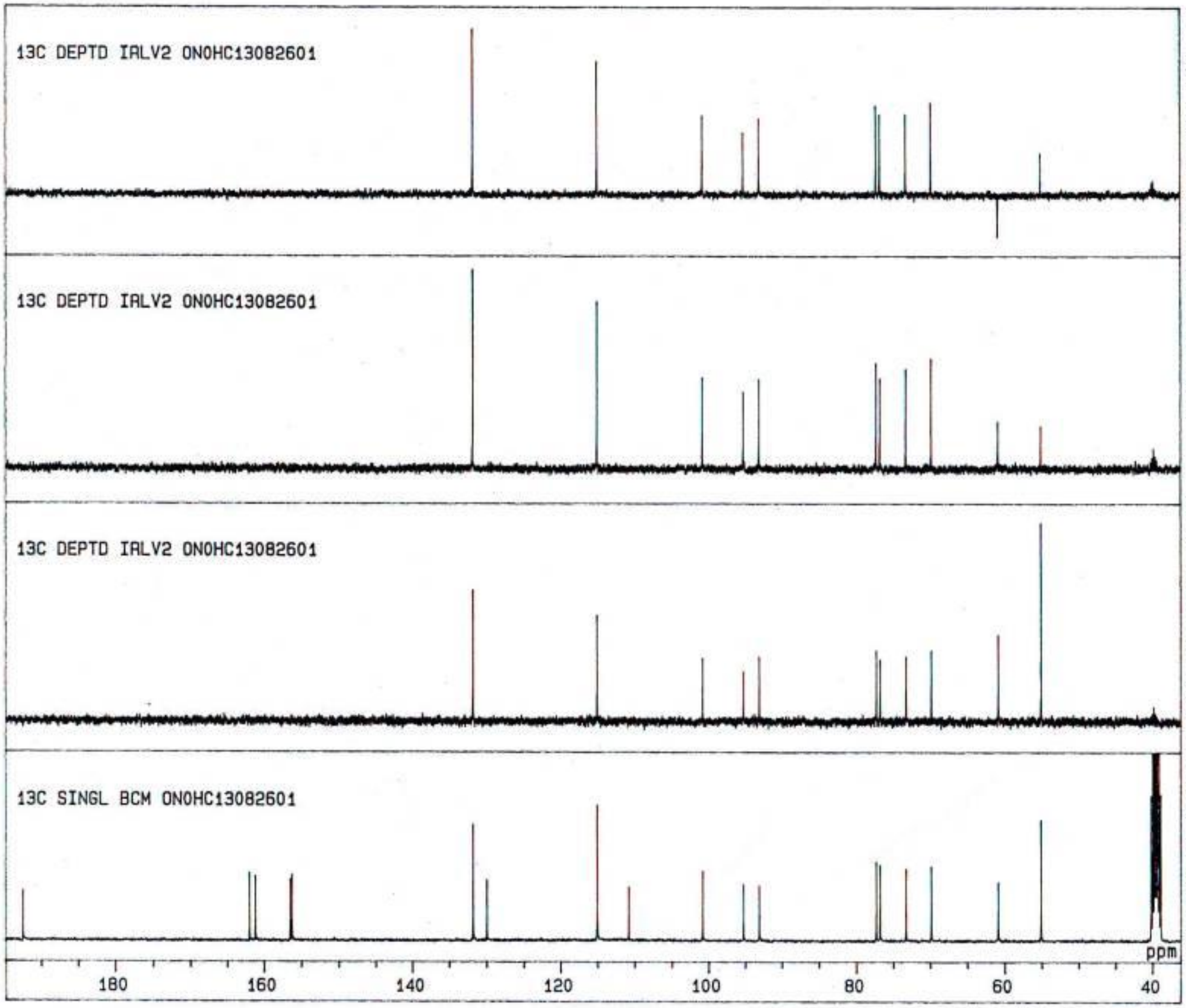

27-AUG-2013 18:57:01.19

DFILE : ON0HC130826013D135
SFILE : ALPHA400

COMNT : 13C DEPTD IRLV2 ON0HC13082[illegible]
EXMOD : DEPTD
IRMOD : IRLV2
POINT : 32768
FREQU : 27100.27 Hz
SCANS : 512
DUMMY : 4
ACQTM : 1.2091 sec
PD : 1.7909 sec
RGAIN : 23

PW1 : 9.60 usec
PW2 : 16.20 usec
PW3 : 10.80 usec
JTIM1 : 5.76923 msec
JCNST : 130.00 Hz

OBNUC : 13C
OBFRQ : 100.40 MHz
OBSET : 135500.00 Hz

IRNUC : 1H
IRFRQ : 399.65 MHz
IRSET : 134500.00 Hz
IRATN : 511
IRRPW : 50.0 usec
IRBP1 : 26
IRBP2 : 6
IRRNS : 0

ADBIT : 16
CTEMP : 30.0 c
CSPED : 13 Hz
SLVNT : DMSO

RESOL : 0.83 Hz
BF : 1.50 Hz
REFVL : 39.50 ppm
XE : 15949.36 Hz
XS : -1551.93 Hz
operator

1H COSY NON ONOHC13082601

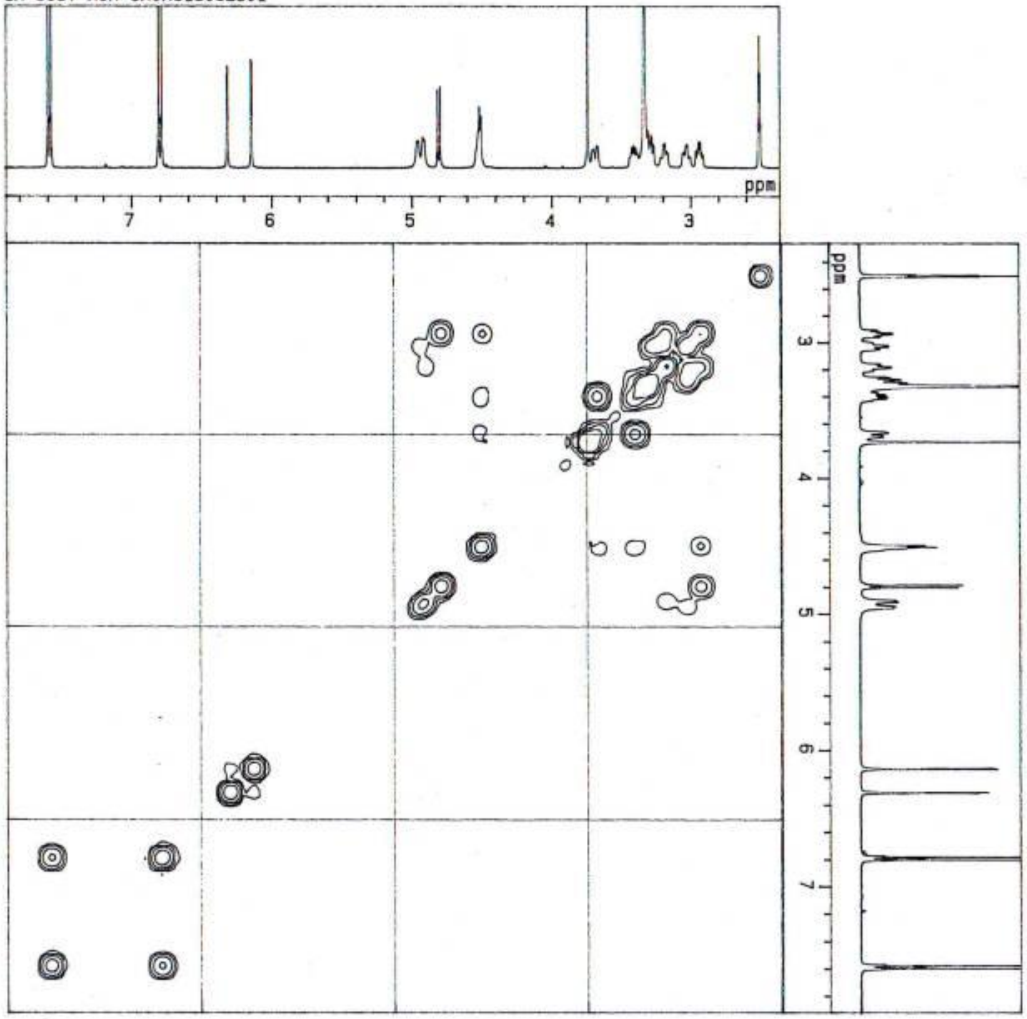

27-AUG-2013 18: 31: 28.23

DFILE : ALPHA
SFILE : ONOHC130826014COSY

COMNT : 1H COSY NON ONOHC13082601
EXMOD : COSY
IRMOD : NON
POINT : 512
FREQU : 4909.18 Hz
SCANS : 64
DUMMY : 4
ACQTM : 0.0521 sec
PD : 0.9479 sec
RGAIN : 13

CLFRQ : 4909.18 Hz
CLPNT : 512
TOSCN : 256
CINWT : 10.00 usec
CINTV : 203.70 usec

PW1 : 10.80 usec
PW2 : 21.60 usec
PI1 : 120.0000 msec
PI2 : 1.0000 msec

OBNUC : 1H
OBFRQ : 399.65 MHz
OBSET : 134547.86 Hz

IRNUC : 1H
IRFRQ : 399.65 MHz
IRSET : 134500.00 Hz
IRATN : 511
IRRPW : 50.0 usec
IRBP1 : 26
IRBP2 : 6
IRRNS : 0

ADBIT : 16
CTEMP : 30.2 c
CSPED : 12 Hz
SLVNT : DMSO

BESOL : 9.59 Hz
CLRSQ : 9.59 Hz
TLINE : 4
THTOP : 13.6190
THBTM : 0.5551
operator

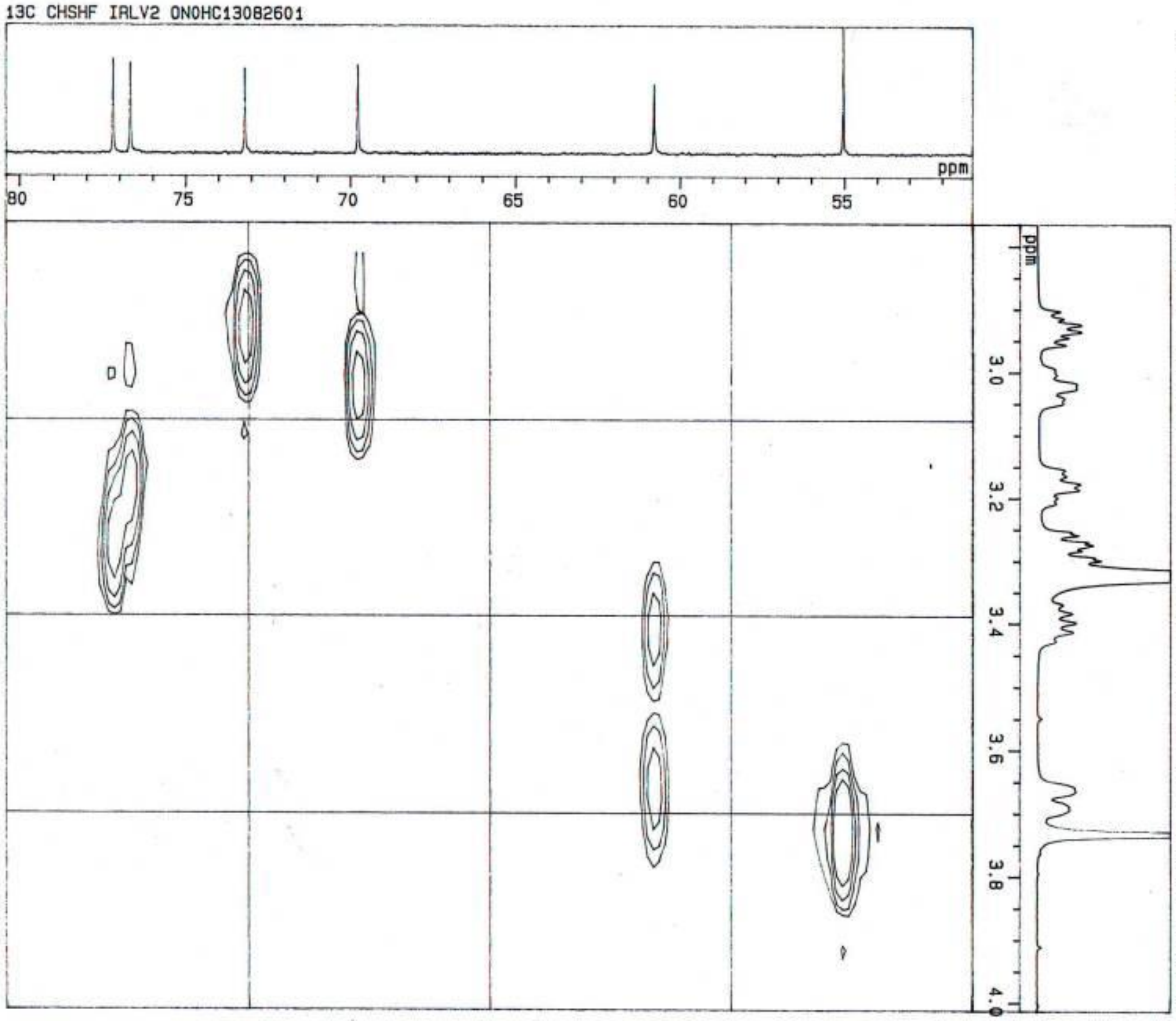

27-AUG-2013 18: 43: 24.40

DFILE : ALPHA
SFILE : ONOHC130826015CHSHF

COMNT : 13C CHSHF IRLV2 ONOHC13082
EXMOD : CHSHF
IRMOD : IRLV2
POINT : 1024
FREQU : 18518.52 Hz
SCANS : 80
DUMMY : 4
ACQTM : 0.0276 sec
PD : 1.4724 sec
RGAIN : 20

CLFRQ : 4908.22 Hz
CLPNT : 256
TOSCN : 128
CINWT : 10.00 usec
CINT2 : 101.87 usec

PW1 : 9.60 usec
PW3 : 10.80 usec
PI1 : 120.0000 msec
PI3 : 5.7128 msec
JCNST : 140.00 Hz

OBNUC : 13C
OBFRQ : 100.40 MHz
OBSET : 137069.71 Hz

IRNUC : 1H
IRFRQ : 399.65 MHz
IRSET : 134547.86 Hz
IRATN : 511
IRRPW : 50.0 usec
IRBP1 : 26
IRBP2 : 6
IRRNS : 0

ADBIT : 16
CTEMP : 30.1 c
CSPED : 13 Hz
SLVNT : DMSO

RESOL : 18.08 Hz
CLRSO : 19.17 Hz
TLINE : 4
THTOP : 4.0063
THBTM : 0.7512
operator

13C CHSHF IRLV2 ONOHC13082601

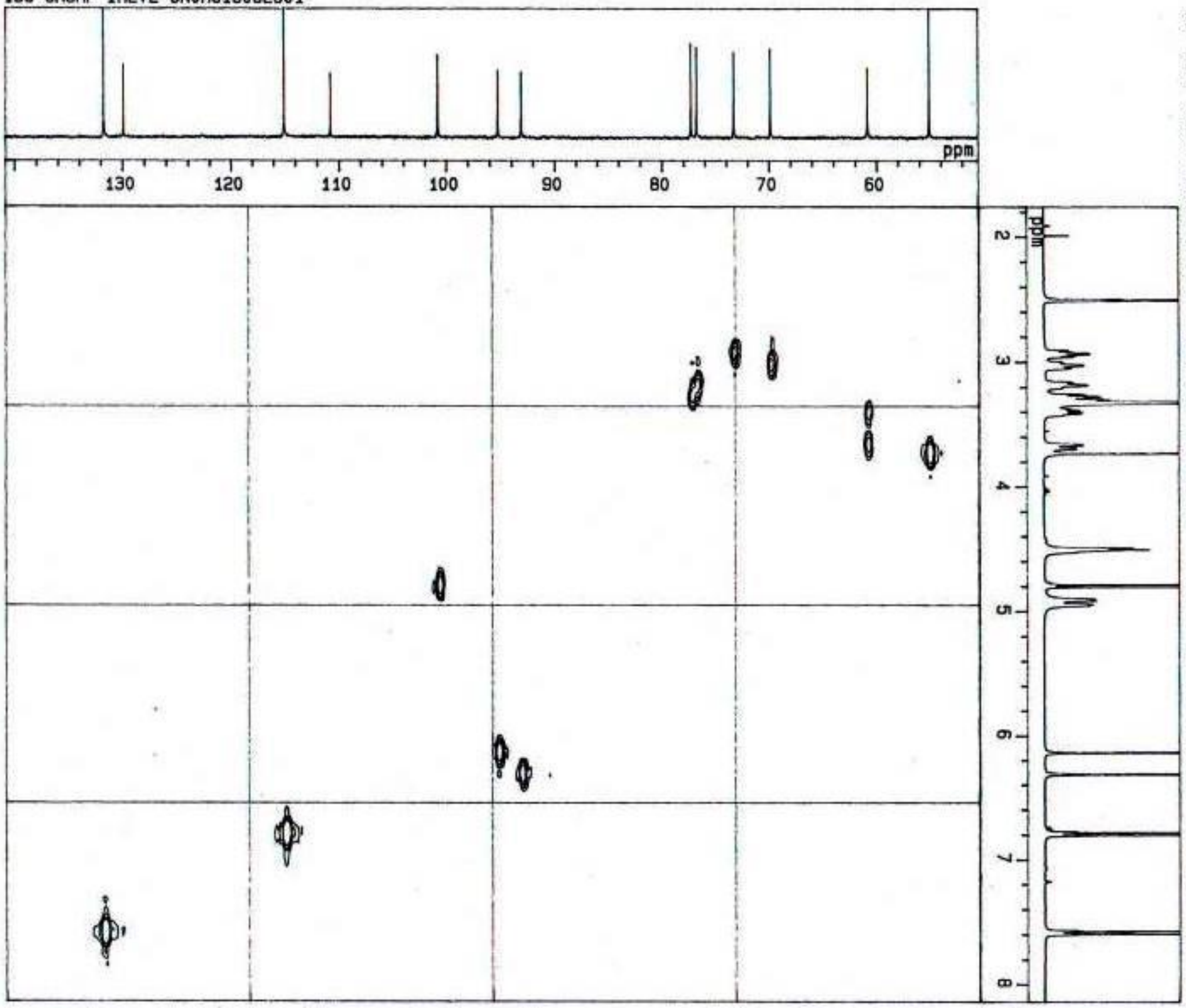

27-AUG-2013 18:39:37.03

DFILE : ALPHA
SFILE : ONOHC130826015CHSHF

COMNT : 13C CHSHF IRLV2 ONOHC130826
EXMOD : CHSHF
IRMOD : IRLV2
POINT : 1024
FREQU : 18518.52 Hz
SCANS : 80
DUMMY : 4
ACQTM : 0.0276 sec
PD : 1.4724 sec
RGAIN : 20

CLFRQ : 4908.22 Hz
CLPNT : 256
TOSCN : 128
CINWT : 10.00 usec
CINT2 : 101.87 usec

PW1 : 9.60 usec
PW3 : 10.80 usec
PI1 : 120.0000 msec
PI3 : 5.7128 msec
JCNST : 140.00 Hz

OBNUC : 13C
OBFRQ : 100.40 MHz
OBSET : 137069.71 Hz

IRNUC : 1H
IRFRQ : 399.65 MHz
IRSET : 134547.86 Hz
IRATN : 511
IRRPW : 50.0 usec
IRBP1 : 26
IRBP2 : 6
IRRNS : 0

ADBIT : 16
CTEMP : 30.1 c
CSPED : 13 Hz
SLVNT : DMSO

RESOL : 18.08 Hz
CLRSO : 19.17 Hz
TLINE : 4
THTOP : 4.0063
THBTM : 0.7512

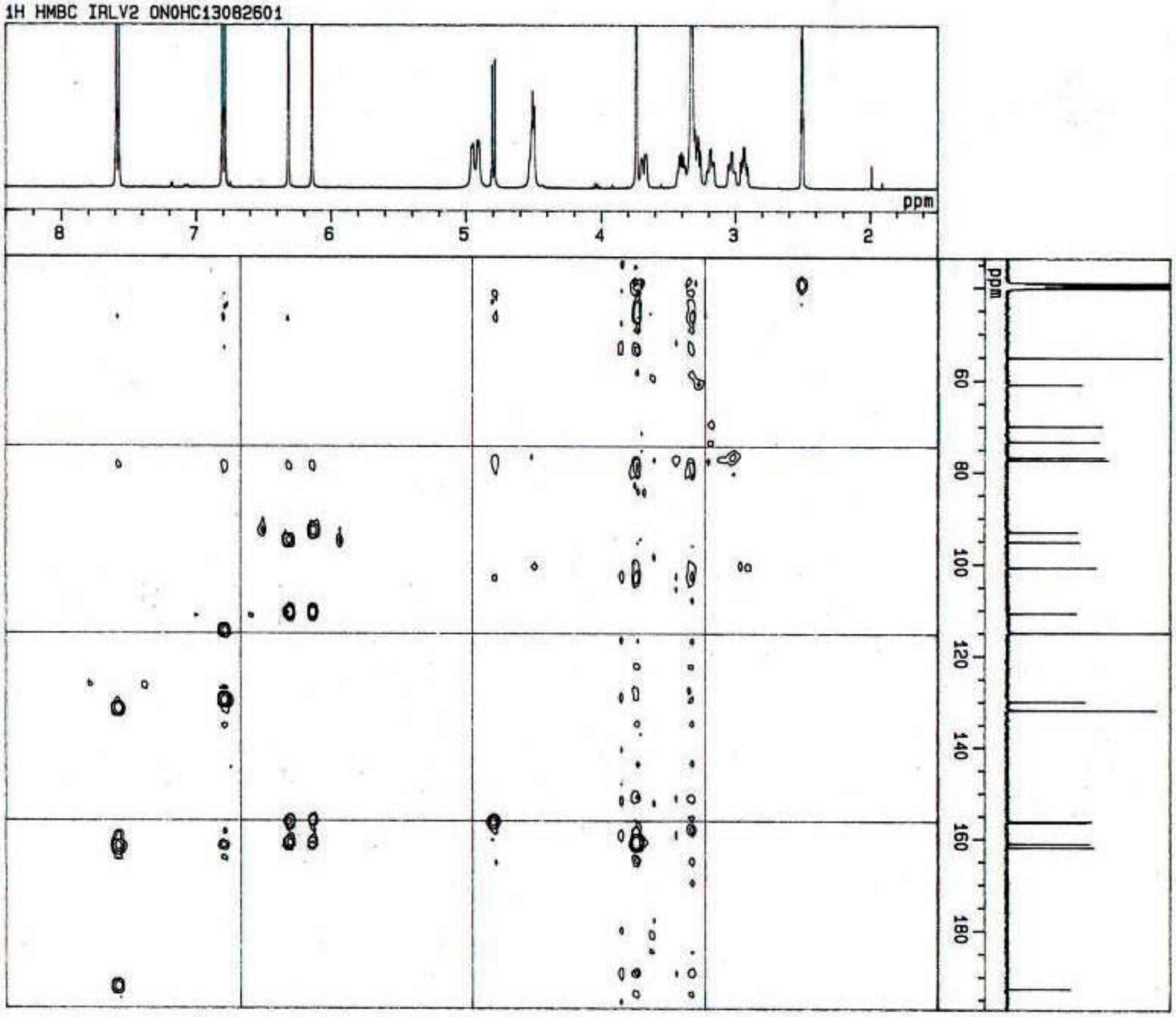
1H HMBC IRLV2 ONOHC13082601
ppm
8
7
6
5
4
3
2
60
80
100
120
140
160
180

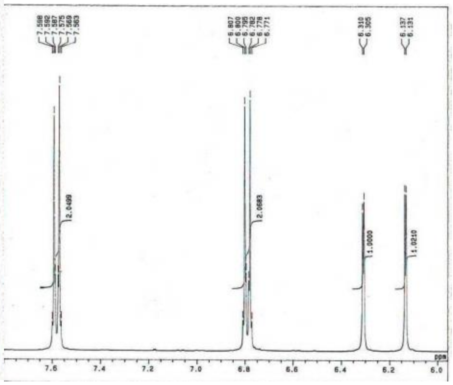

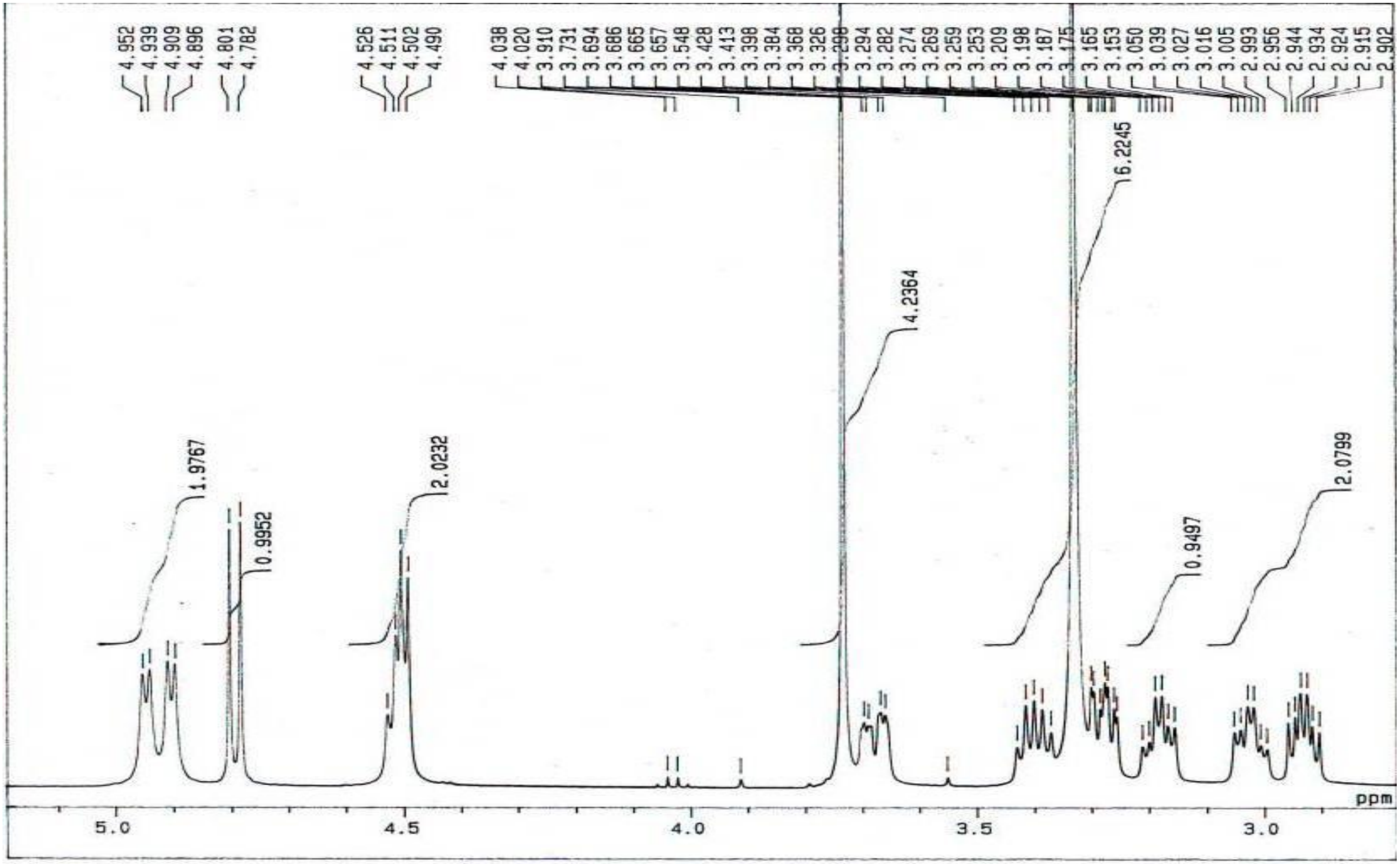

4.952
4.939
4.909
4.896
4.801
4.782
4.526
4.511
4.502
4.490
4.038
4.020
3.910
3.731
3.694
3.686
3.665
3.657
3.548
3.428
3.413
3.398
3.384
3.368
3.326
3.298
3.294
3.282
3.274
3.269
3.259
3.253
3.209
3.198
3.187
3.175
3.165
3.153
3.050
3.039
3.027
3.016
3.005
2.993
2.956
2.944
2.934
2.924
2.915
2.902
1.9767
0.9952
2.0232
4.2364
6.2245
0.9497
2.0799
5.0
4.5
4.0
3.5
3.0
ppm

Compound 2

IR spectra

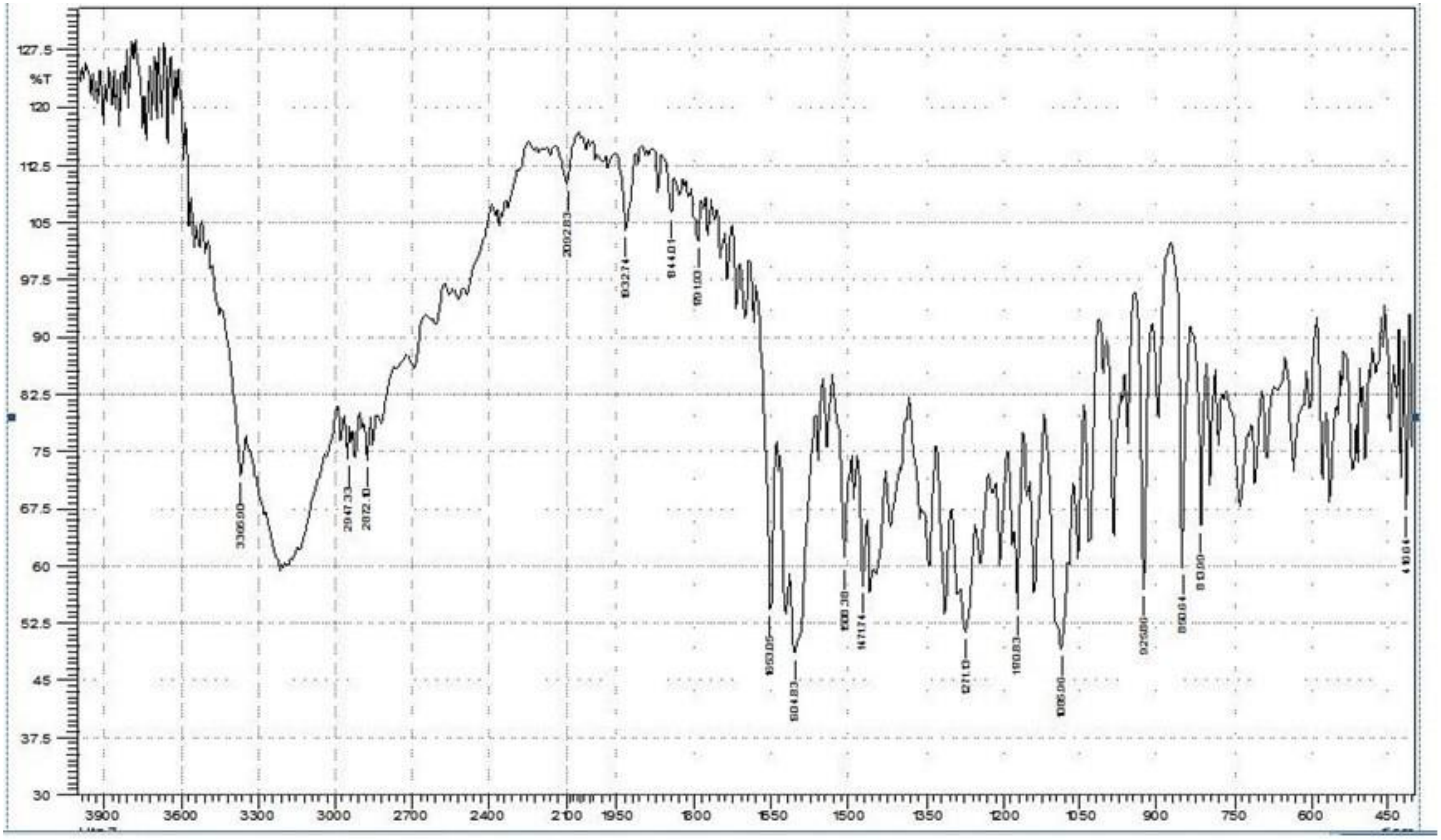

LCMS Spectra

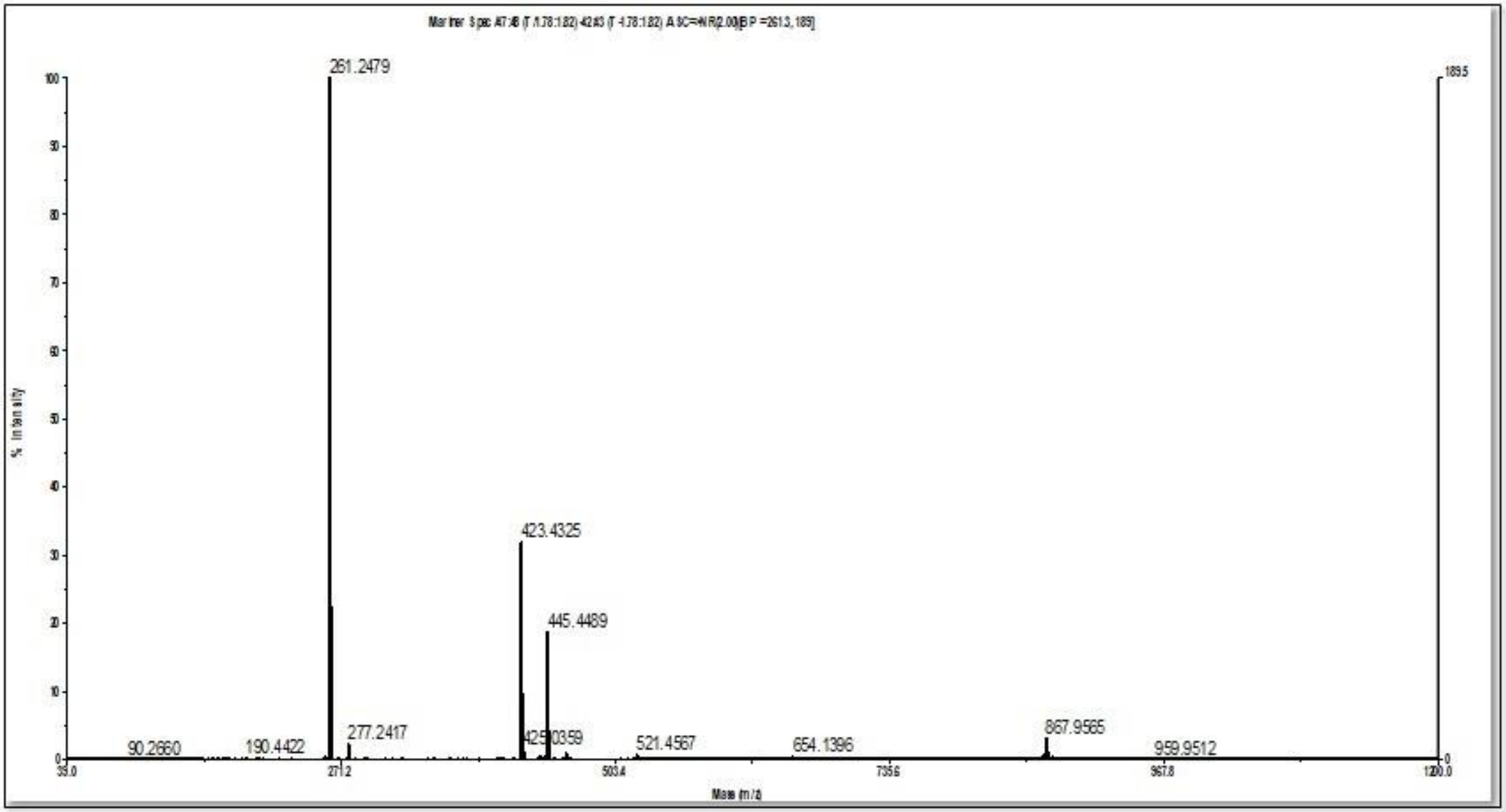

NMR spectra, HMBC and HSQC

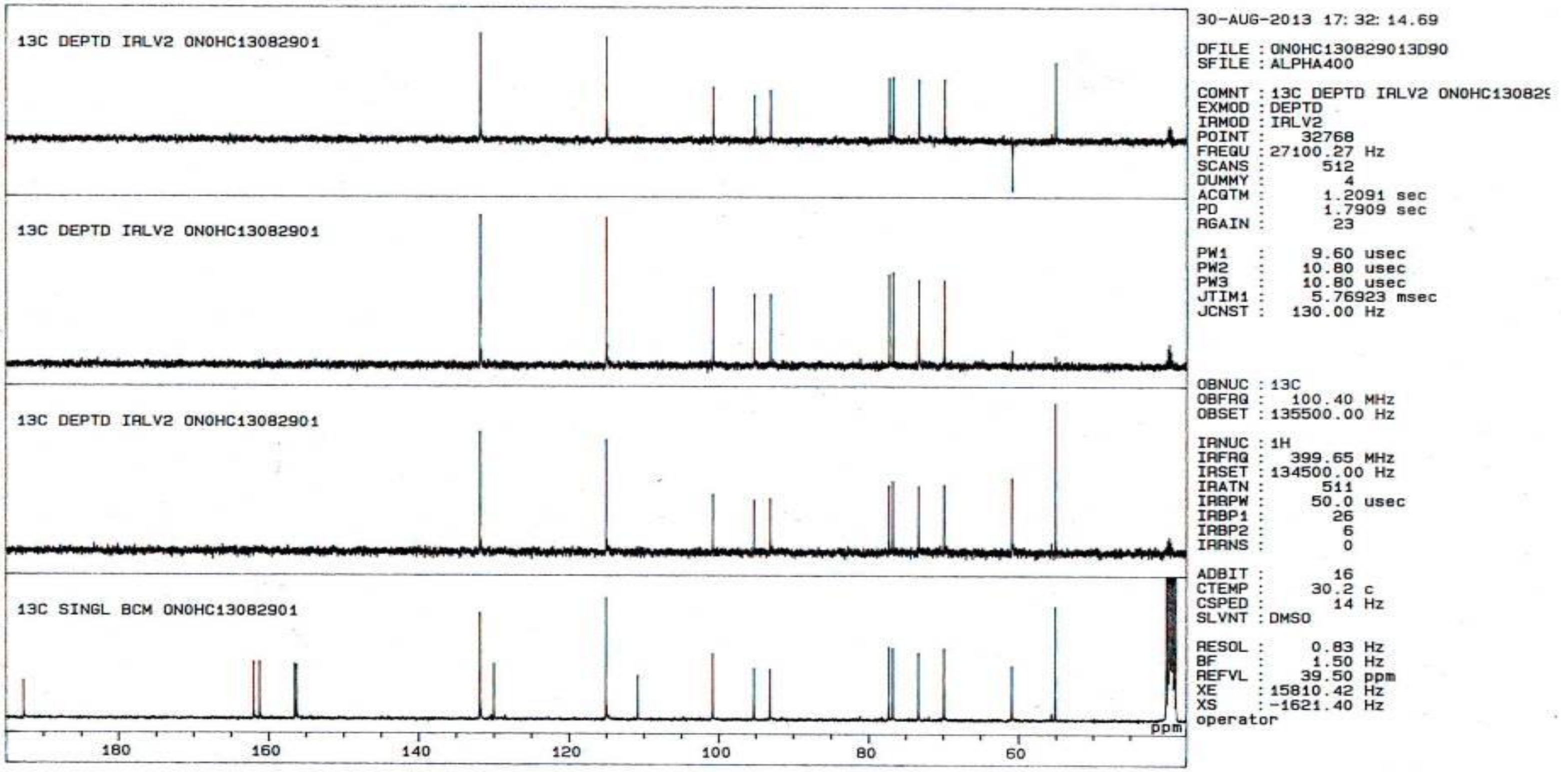

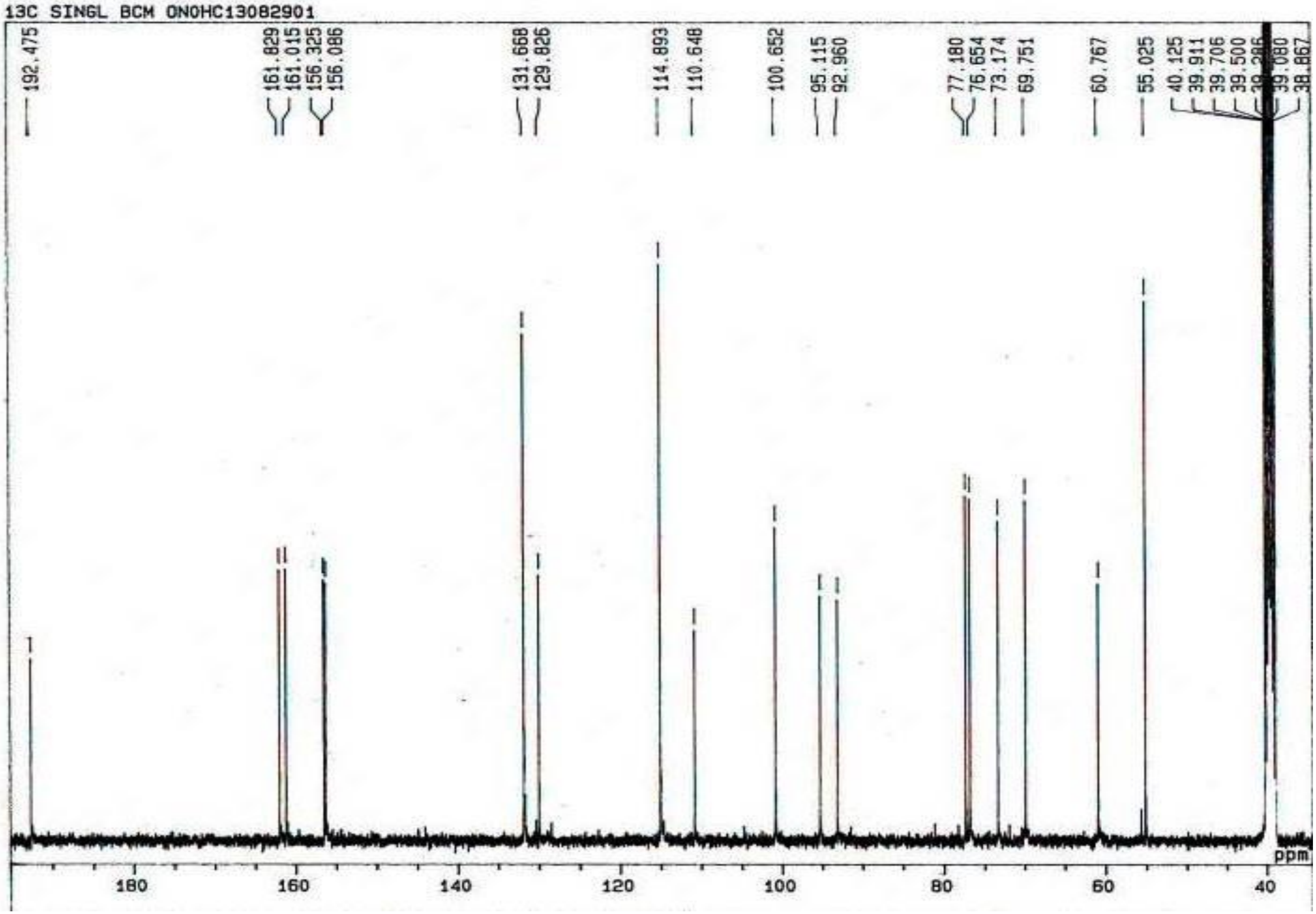
13C SINGL BCM ON0HC13082901
192.475
161.829
161.015
156.325
156.086
131.668
129.826
114.893
110.648
100.652
95.115
92.960
77.180
76.654
73.174
69.751
60.767
55.025
40.125
39.911
39.706
39.500
39.080
38.867
ppm
180
160
140
120
100
80
60
40

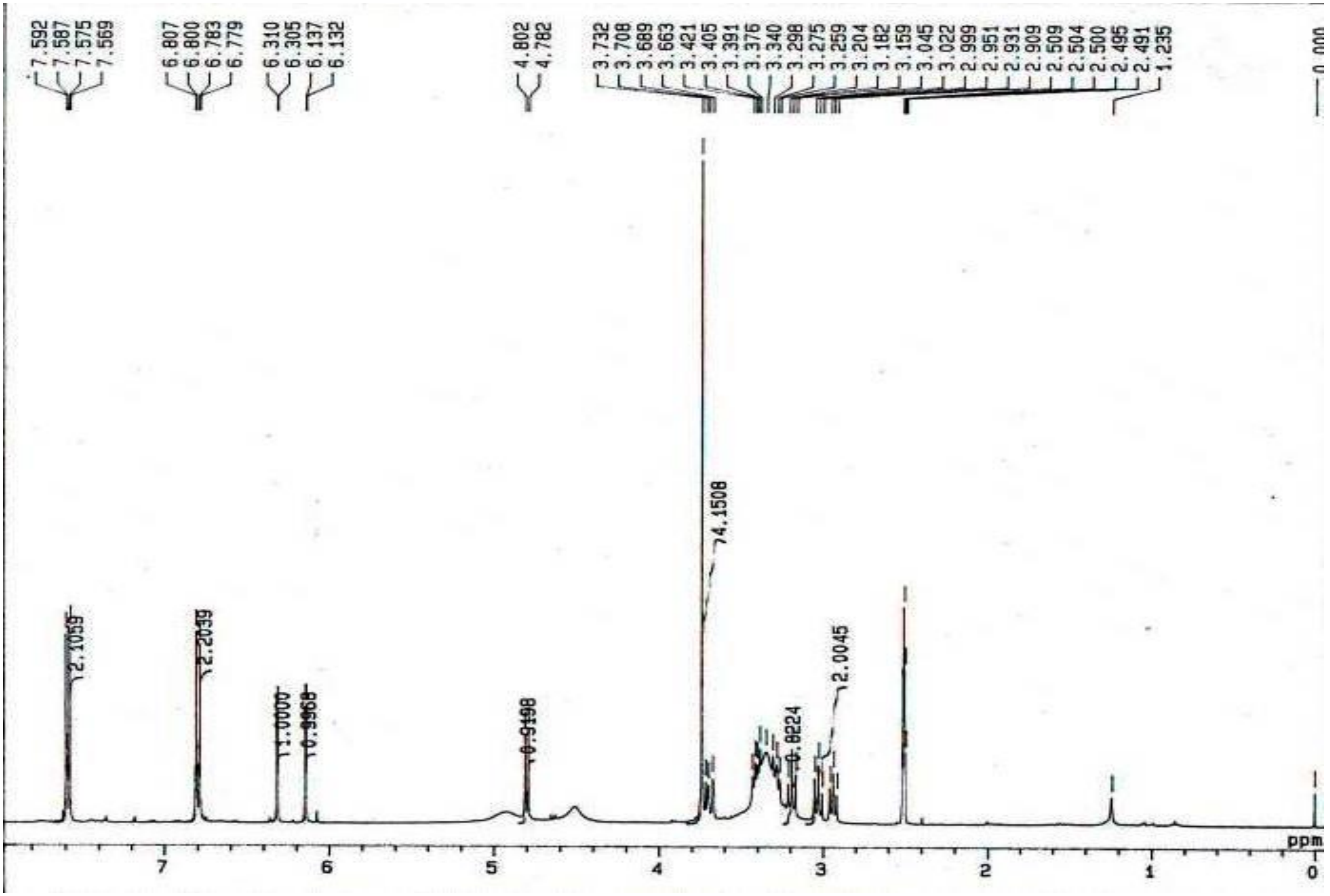

7.592
7.587
7.575
7.569
6.807
6.800
6.783
6.779
6.310
6.305
6.137
6.132
4.802
4.782
3.732
3.708
3.689
3.663
3.421
3.405
3.391
3.376
3.340
3.298
3.275
3.259
3.204
3.182
3.159
3.045
3.022
2.999
2.951
2.931
2.909
2.509
2.504
2.500
2.495
2.491
1.235
0.000
2.1059
2.2039
1.0000
0.9968
0.9198
4.1508
0.8224
2.0045
ppm
7
6
5
4
3
2
1
0

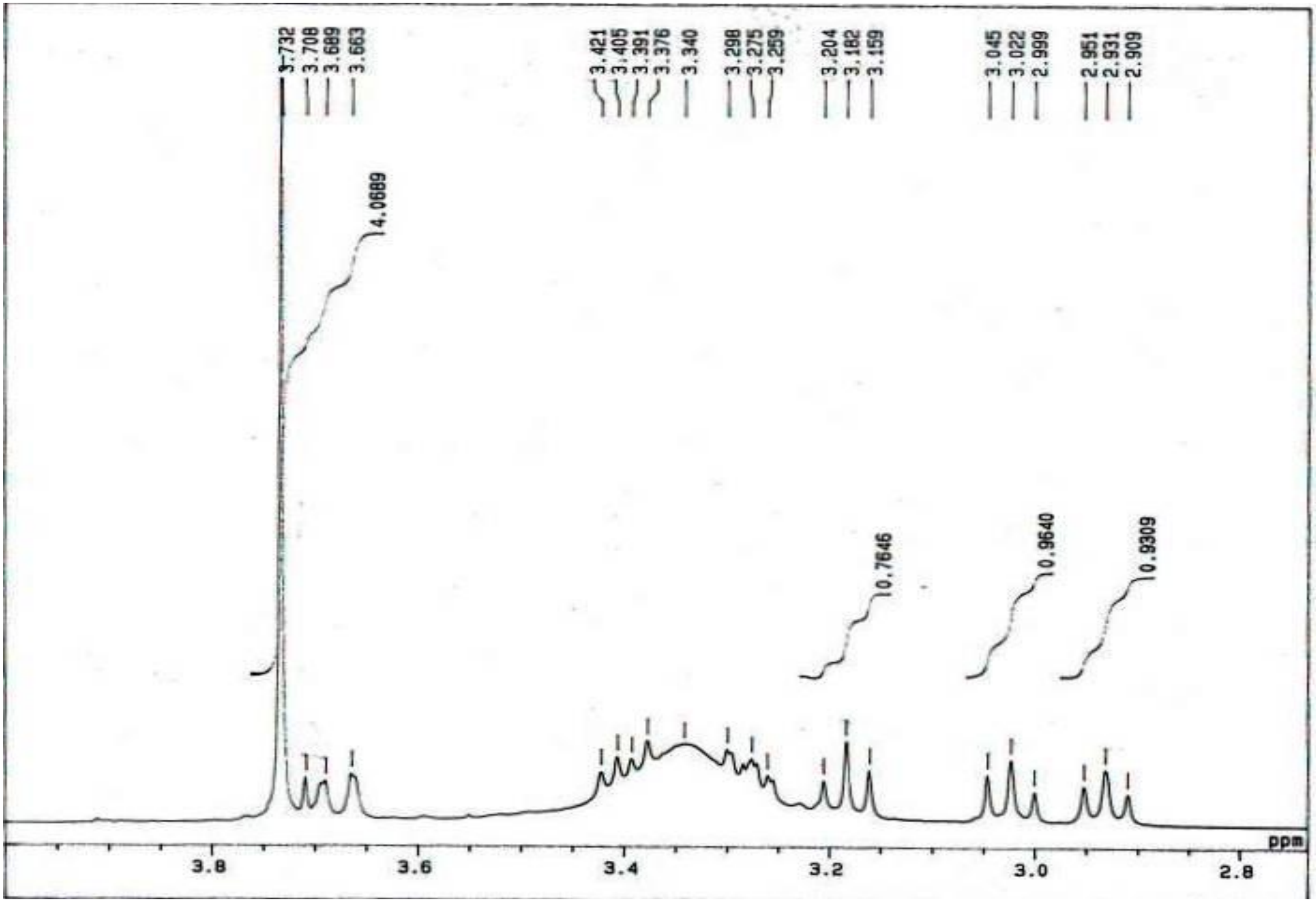

3.732
3.708
3.689
3.663
3.421
3.405
3.391
3.376
3.340
3.298
3.275
3.259
3.204
3.182
3.159
3.045
3.022
2.999
2.951
2.931
2.909
4.0689
0.7646
0.9640
0.9309
3.8
3.6
3.4
3.2
3.0
2.8
ppm

1H HMBC IRLV2 ON0HC13082901

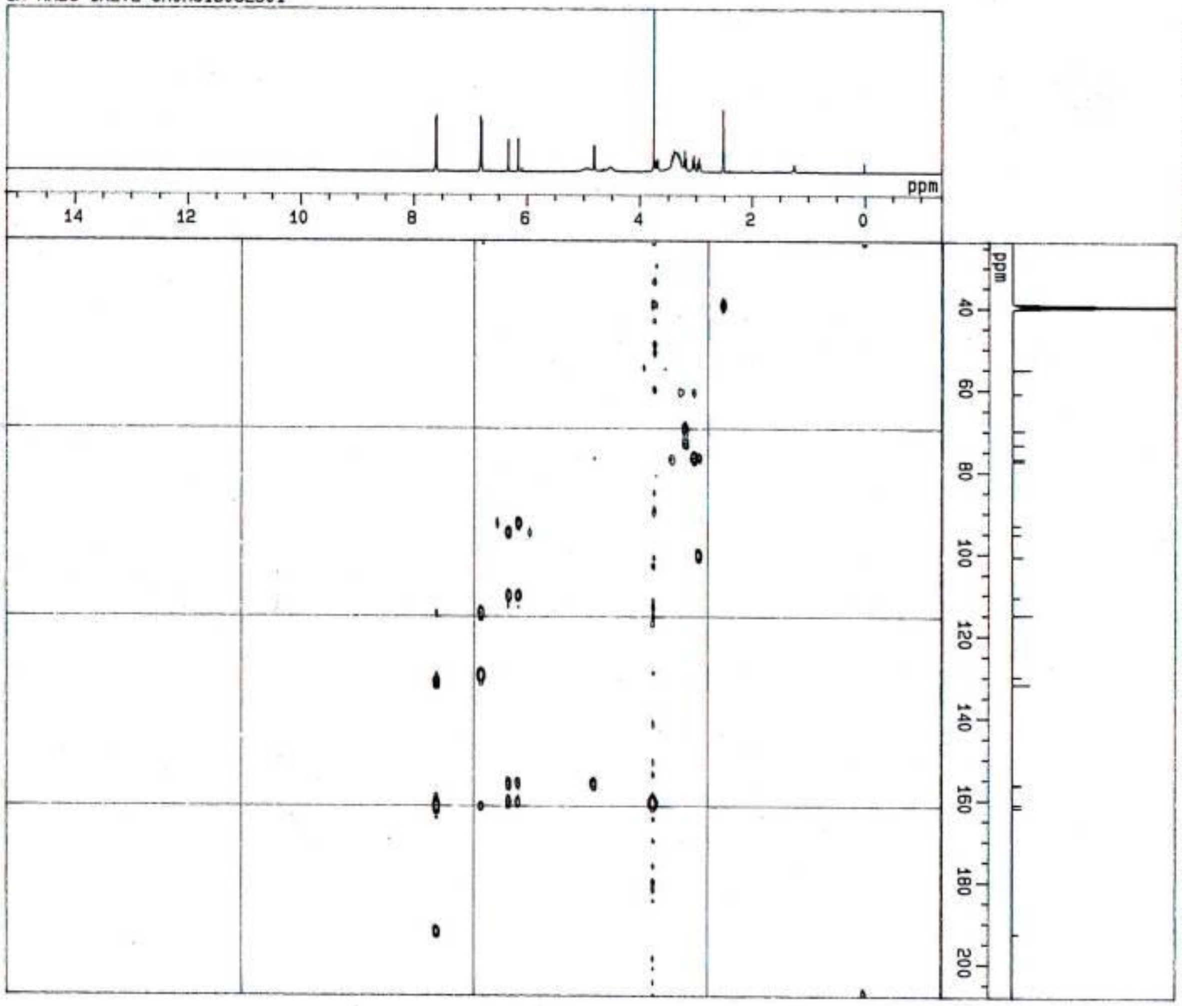

30-AUG-2013 16:55:09.02

DFILE : ON0HC130829016HMBC
SFILE : ALPHA400

COMNT : 1H HMBC IRLV2 ON0HC1308290:
EXMOD : HMBC
IRMOD : IRLV2
POINT : 512
FREQU : 6622.52 Hz
SCANS : 80
DUMMY : 32
ACQTM : 0.0773 sec
PD : 1.4227 sec
RGAIN : 14

CLFRQ : 18532.25 Hz
CLPNT : 256
TOSCN : 128
CINWT : 10.00 usec
CINT2 : 26.98 usec

PW1 : 10.80 usec
PW3 : 9.60 usec
PI1 : 62.5000 msec
PI3 : 74.8544 msec
JCNST : 140.00 Hz

OBNUC : 1H
OBFRQ : 399.65 MHz
OBSET : 135261.87 Hz

IRNUC : 13C
IRFRQ : 100.40 MHz
IRSET : 137069.71 Hz
IRATN : 511
IRRPW : 48.0 usec
IRBP1 : 50
IRBP2 : 6
IRRNS : 0

ADBIT : 16
CTEMP : 30.2 c
CSPED : 0 Hz
SLVNT : DMSO

RESOL : 12.93 Hz
CLRSO : 72.39 Hz
TLINE : 4
THTOP : 0.2328
THBTM : 0.0222
operator

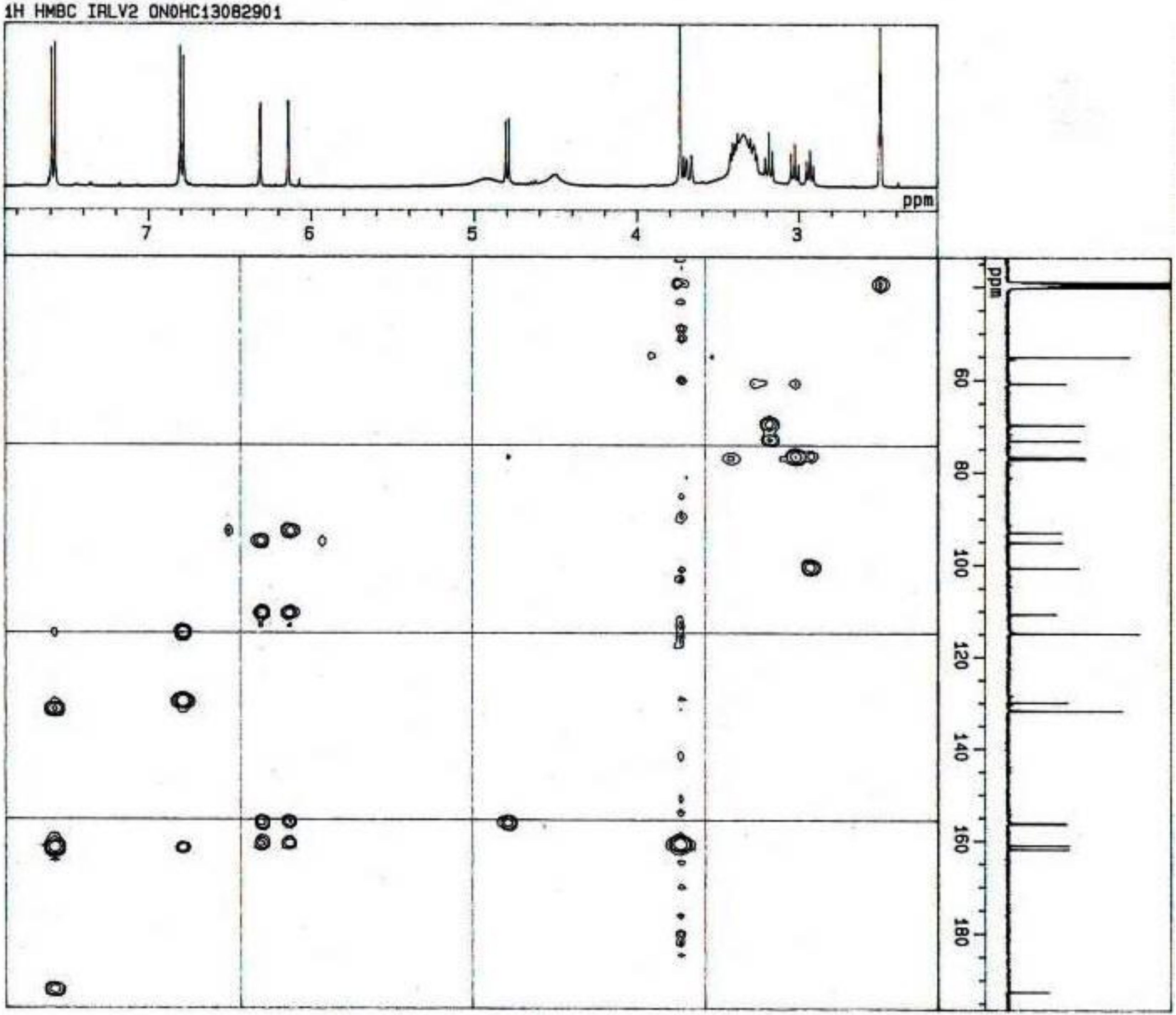
1H HMBC IRLV2 ONOHC13082901
ppm
7
6
5
4
3
ppm
60
80
100
120
140
160
180

13C CHSHF IRLV2 ONOHC13082901

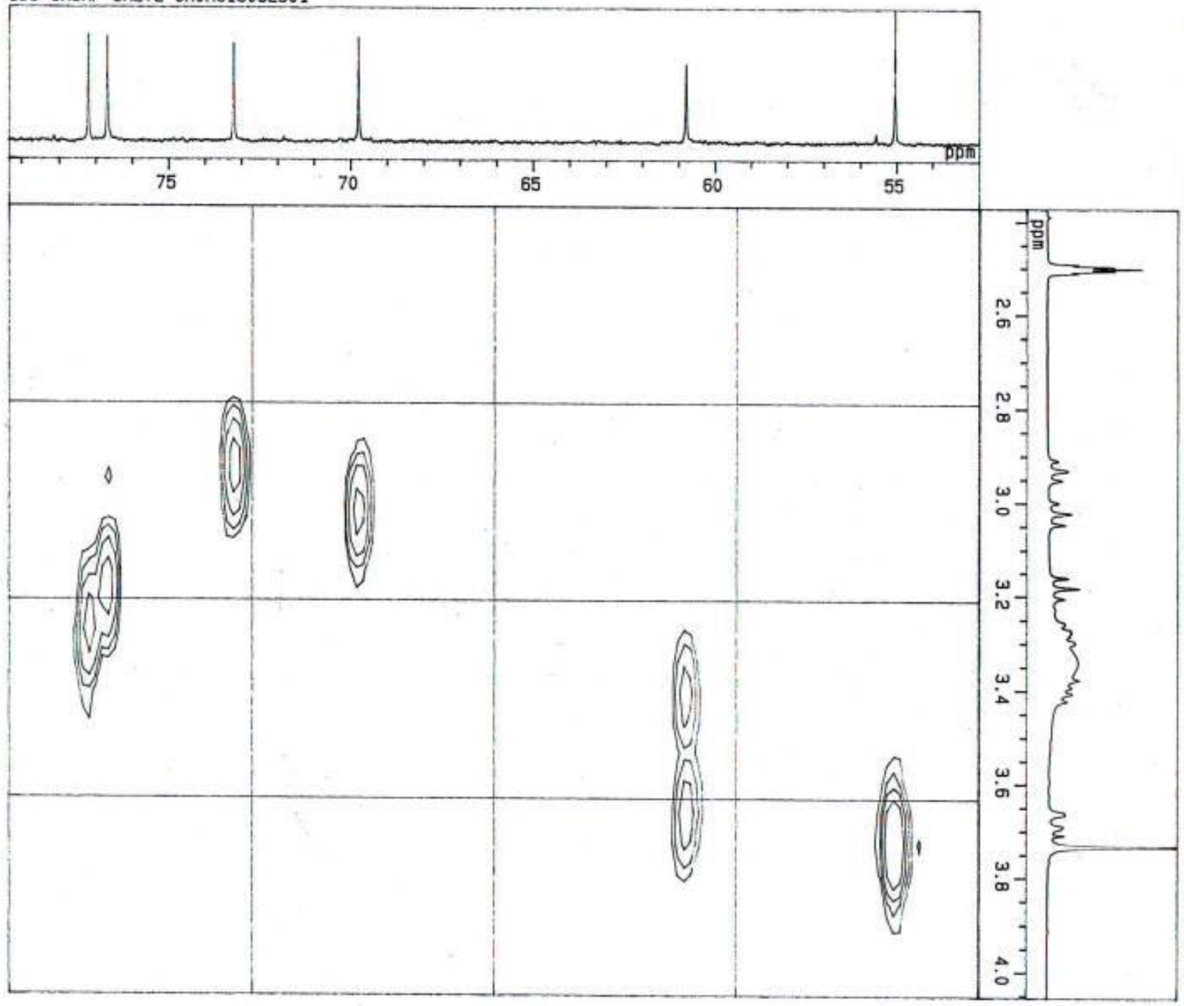

30-AUG-2013 17:52:26.14

DFILE : ALPHA
SFILE : ONOHC130829015CHSHF

COMNT : 13C CHSHF IRLV2 ONOHC13082[illegible]
EXMOD : CHSHF
IRMOD : IRLV2
POINT : 1024
FREQU : 18518.52 Hz
SCANS : 80
DUMMY : 4
ACQTM : 0.0276 sec
PD : 1.4724 sec
RGAIN : 20

CLFRQ : 6622.52 Hz
CLPNT : 256
TOSCN : 128
CINWT : 10.00 usec
CINT2 : 75.50 usec

PW1 : 9.60 usec
PW3 : 10.80 usec
PI1 : 120.0000 msec
PI3 : 5.7128 msec
JCNST : 140.00 Hz

OBNUC : 13C
OBFRQ : 100.40 MHz
OBSET : 137069.71 Hz

IRNUC : 1H
IRFRQ : 399.65 MHz
IRSET : 135261.87 Hz
IRATN : 511
IRRPW : 50.0 usec
IRBP1 : 26
IRBP2 : 6
IRRNS : 0

ADBIT : 16
CTEMP : 30.2 c
CSPED : 13 Hz
SLVNT : DMSO

RESOL : 18.08 Hz
CLRSO : 25.87 Hz
TLINE : 4
THTOP : 4.2113
THBTM : 0.9384
operator

13C CHSHF IRLV2 ON0HC13082901

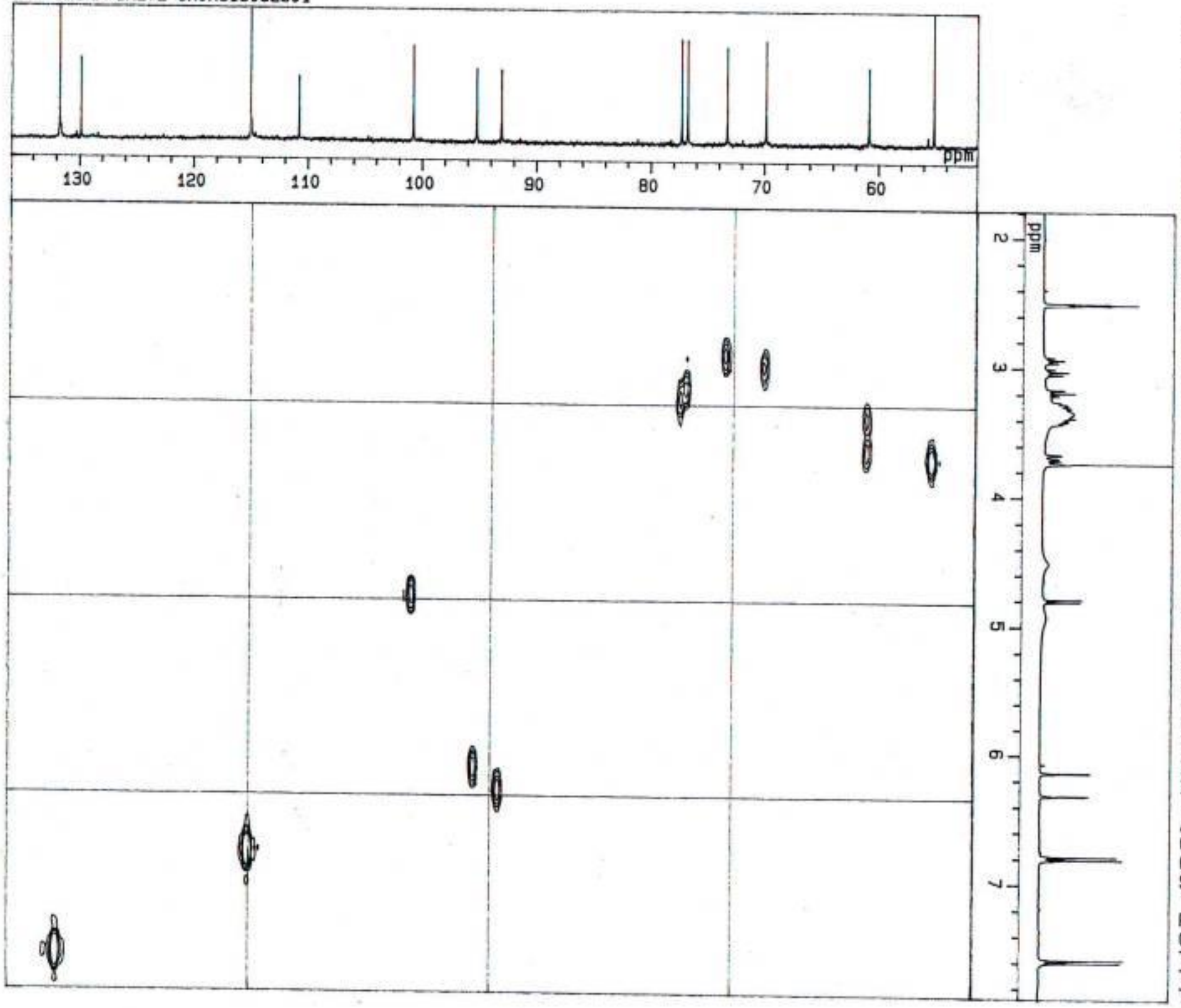

30-AUG-2013 17: 49: 04.66

DFILE : ALPHA
SFILE : ON0HC130829015CHSHF

COMNT : 13C CHSHF IRLV2 ON0HC13082[illegible]
EXMOD : CHSHF
IRMOD : IRLV2
POINT : 1024
FREQU : 18518.52 Hz
SCANS : 80
DUMMY : 4
ACQTM : 0.0276 sec
PD : 1.4724 sec
RGAIN : 20

CLFRQ : 6622.52 Hz
CLPNT : 256
TOSCN : 128
CINWT : 10.00 usec
CINT2 : 75.50 usec

PW1 : 9.60 usec
PW3 : 10.80 usec
PI1 : 120.0000 msec
PI3 : 5.7128 msec
JCNST : 140.00 Hz

OBNUC : 13C
OBFRQ : 100.40 MHz
OBSET : 137069.71 Hz

IRNUC : 1H
IRFRQ : 399.65 MHz
IRSET : 135261.87 Hz
IRATN : 511
IRRPW : 50.0 usec
IRBP1 : 26
IRBP2 : 6
IRRNS : 0

ADBIT : 16
CTEMP : 30.2 c
CSPED : 13 Hz
SLVNT : DMSO

RESOL : 18.08 Hz
CLRSO : 25.87 Hz
TLINE : 4
THTOP : 4.2113
THBTM : 0.9384
operator

1H COSY NON ON0HC13082901

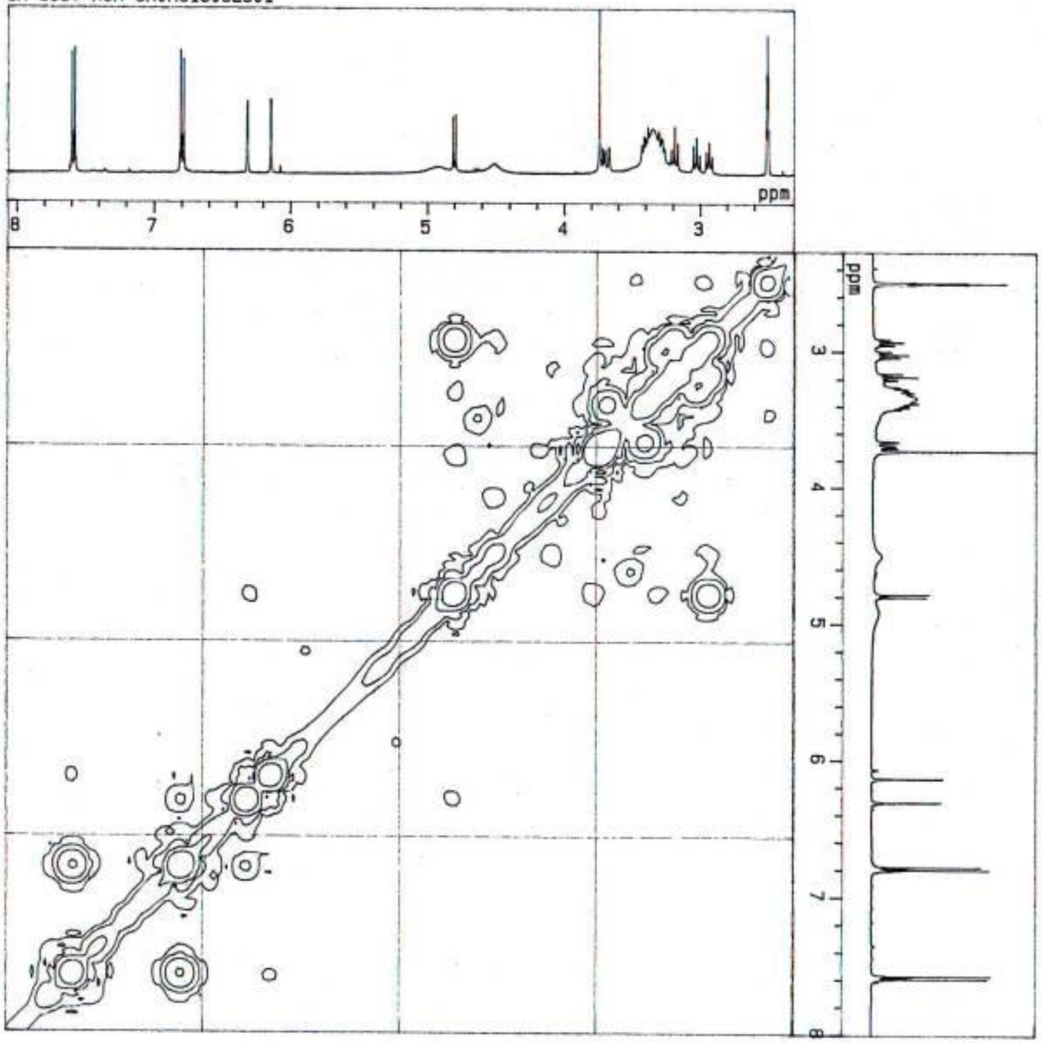

30-AUG-2013 17: 42: 29.66

DFILE : ALPHA
SFILE : ON0HC130829014COSY

COMNT : 1H COSY NON ON0HC13082901
EXMOD : COSY
IRMOD : NON
POINT : 512
FREQU : 6622.52 Hz
SCANS : 64
DUMMY : 4
ACQTM : 0.0387 sec
PD : 0.9613 sec
RGAIN : 14

CLFRQ : 6622.52 Hz
CLPNT : 512
TOSCN : 256
CINWT : 10.00 usec
CINTV : 151.00 usec

PW1 : 10.80 usec
PW2 : 21.60 usec
PI1 : 120.0000 msec
PI2 : 1.0000 msec

OBNUC : 1H
OBFRQ : 399.65 MHz
OBSET : 135261.87 Hz

IRNUC : 1H
IRFRQ : 399.65 MHz
IRSET : 134500.00 Hz
IRATN : 511
IRRPW : 50.0 usec
IRBP1 : 26
IRBP2 : 6
IRRNS : 0

ADBIT : 16
CTEMP : 30.1 c
CSPED : 14 Hz
SLVNT : DMSO

RESOL : 12.93 Hz
CLRSO : 12.93 Hz
TLINE : 4
THTOP : 11.9106
THBTM : 0.0971
operator

Compound 3: IR spectra

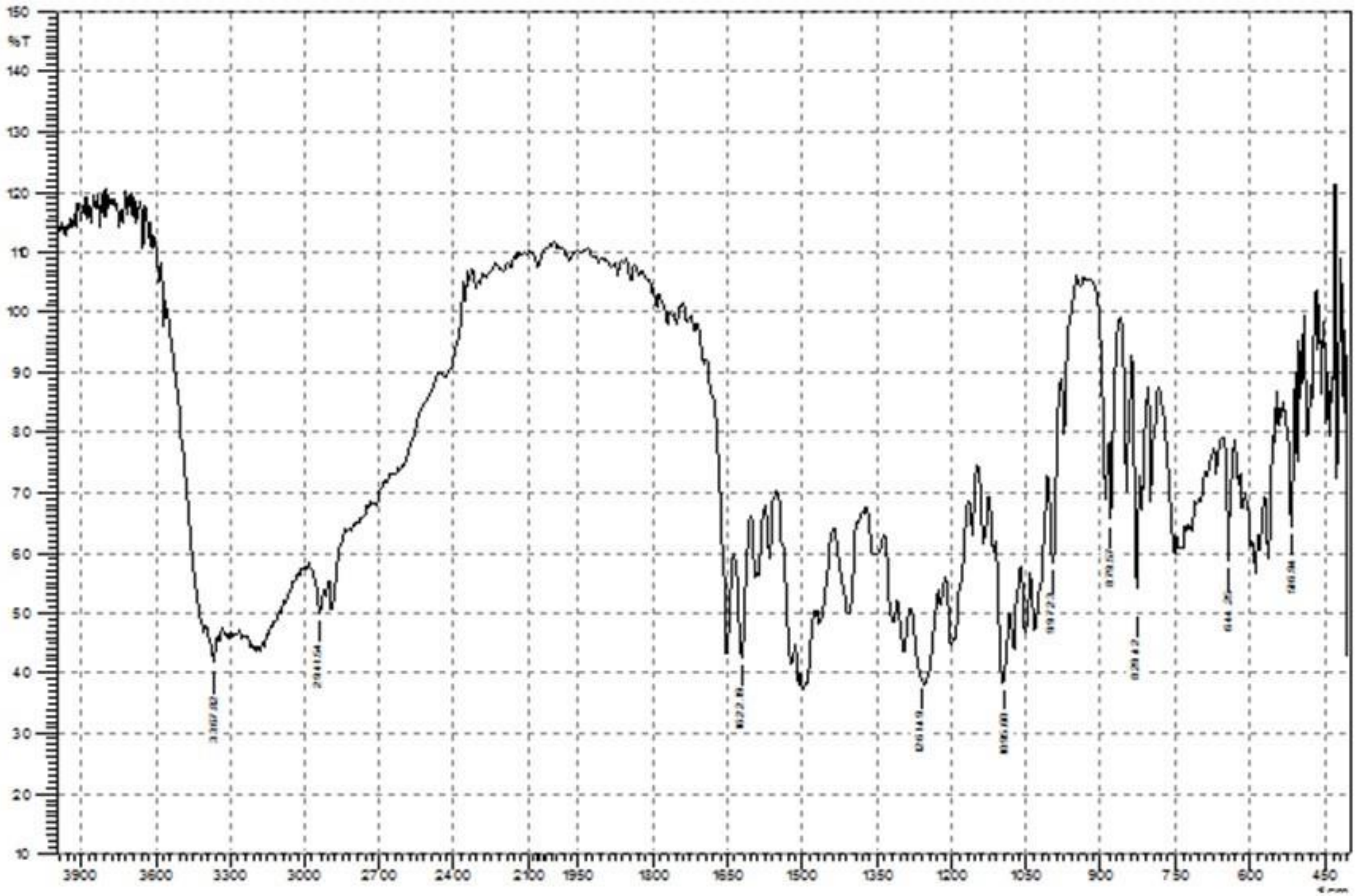

LCMS spectra

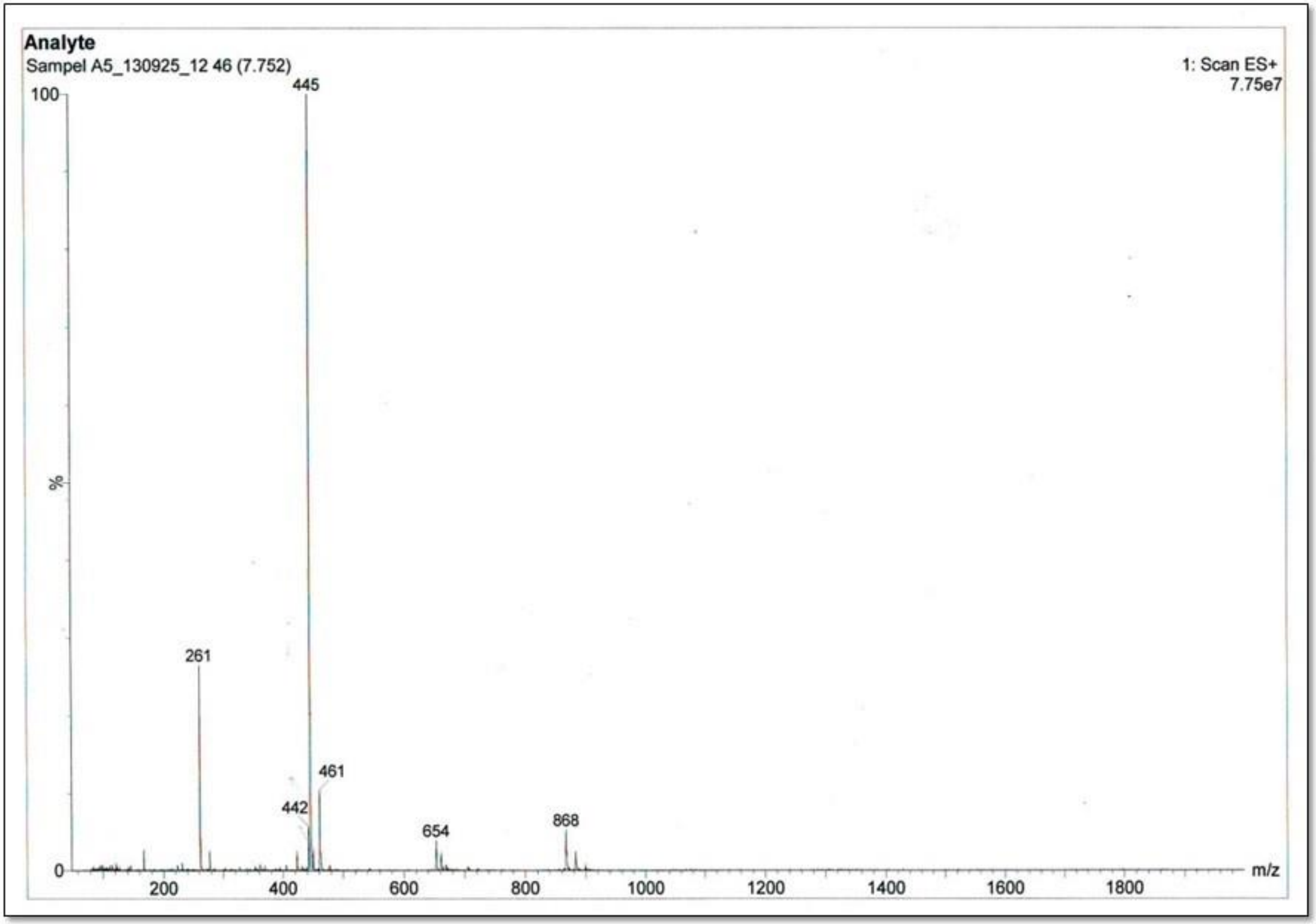

NMR, HMBC and HMQC Spectra

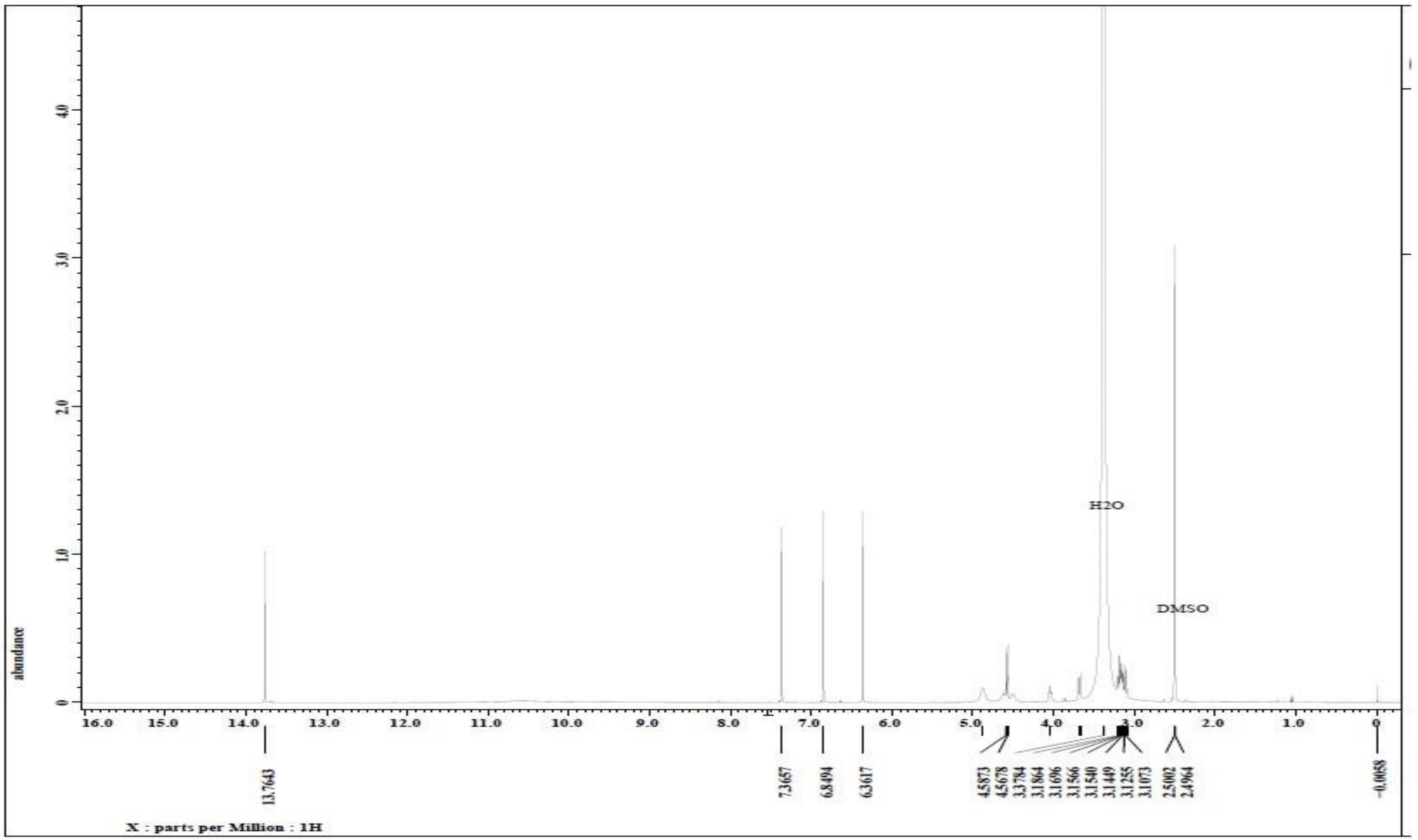

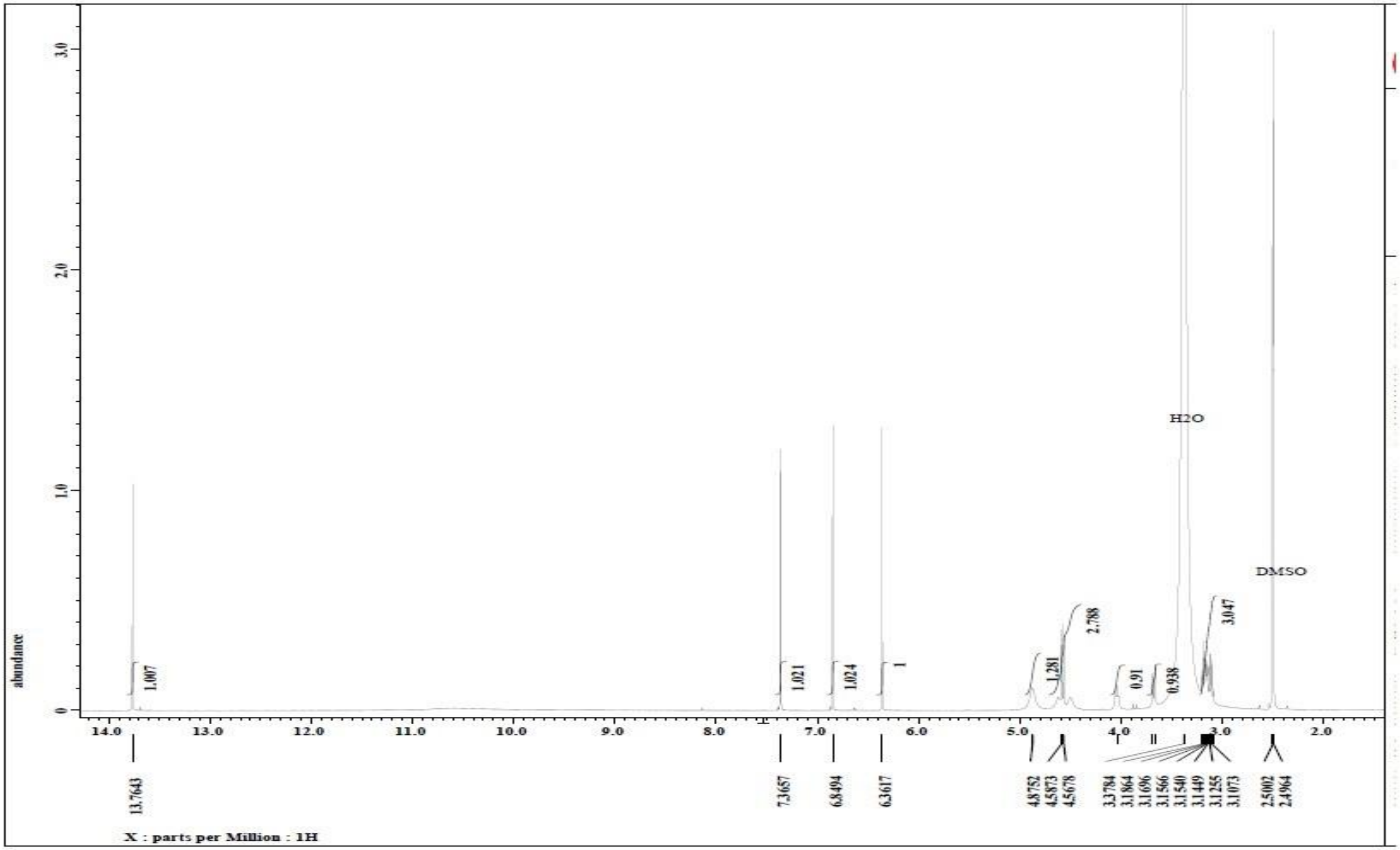

abundance
3.0
2.0
1.0
0
H2O
DMSO
1.007
1.021
1.024
1
1.281
2.788
0.91
0.938
3.047
14.0
13.0
12.0
11.0
10.0
9.0
8.0
7.0
6.0
5.0
4.0
3.0
2.0
13.7643
7.3657
6.8494
6.3617
4.8752
4.5873
4.5678
3.3784
3.1864
3.1696
3.1566
3.1540
3.1449
3.1255
3.1073
2.5002
2.4964
X : parts per Million : 1H

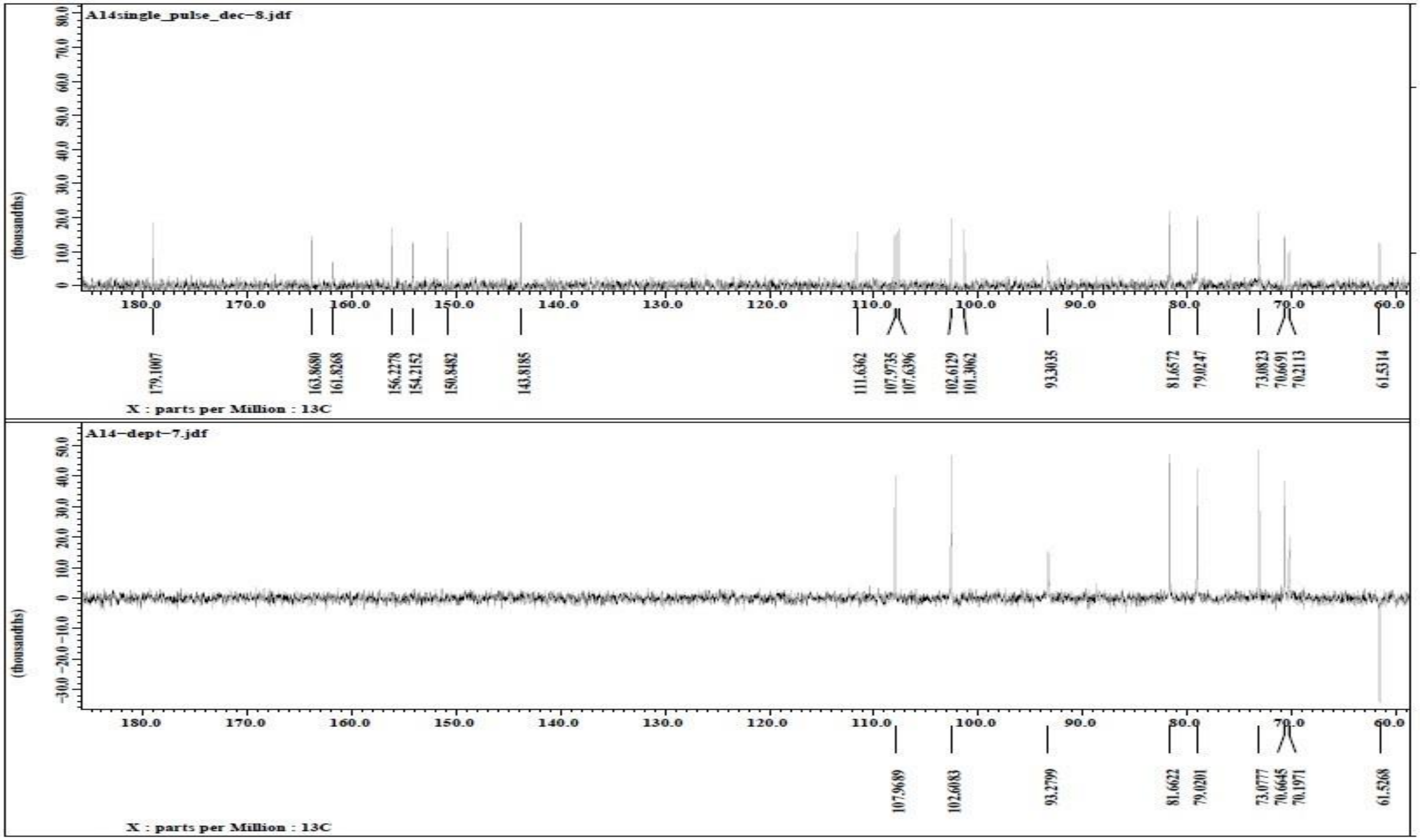

A14single_pulse_dec-8.jdf
(thousandths)
179.1007
163.8680
161.8268
156.2278
154.2152
150.8482
143.8185
111.6362
107.9735
107.6396
102.6129
101.3062
93.3035
81.6572
79.0247
73.0823
70.6691
70.2113
61.5314
X : parts per Million : 13C
A14-dept-7.jdf
(thousandths)
107.9689
102.6083
93.2799
81.6622
79.0201
73.0777
70.6645
70.1971
61.5268
X : parts per Million : 13C

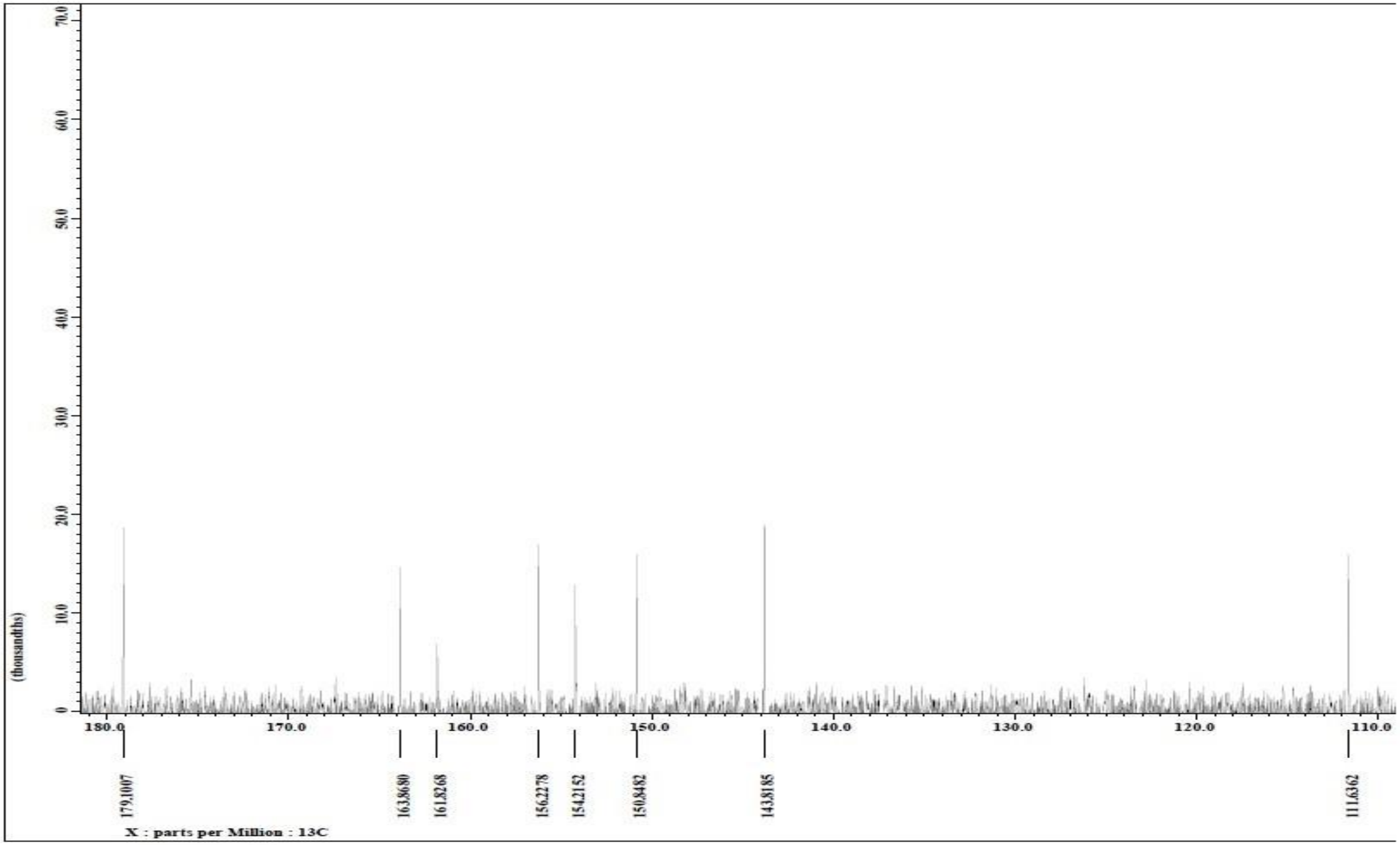

(thousandths)
70.0
60.0
50.0
40.0
30.0
20.0
10.0
0
180.0
170.0
160.0
150.0
140.0
130.0
120.0
110.0
179.1007
163.8680
161.8268
156.2278
154.2152
150.8482
143.8185
111.6362
X : parts per Million : 13C

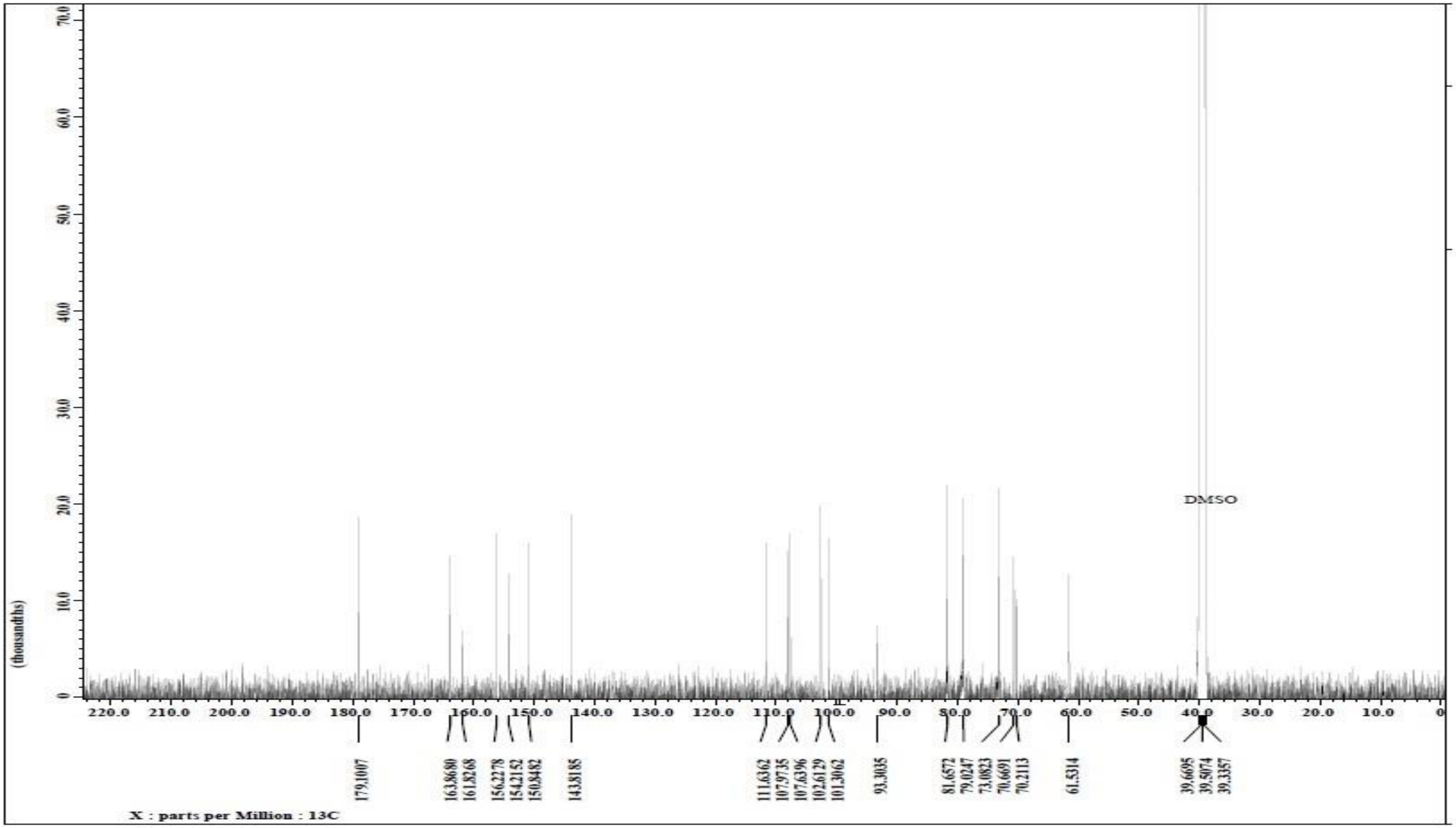

(thousandths)
70.0
60.0
50.0
40.0
30.0
20.0
10.0
0
DMSO
220.0 210.0 200.0 190.0 180.0 170.0 160.0 150.0 140.0 130.0 120.0 110.0 100.0 90.0 80.0 70.0 60.0 50.0 40.0 30.0 20.0 10.0 0
179.1007
163.8680
161.8268
156.2278
154.2152
150.8482
143.8185
111.6362
107.9735
107.6396
102.6129
101.3062
93.3035
81.6572
79.0247
73.0823
70.6691
70.2113
61.5314
39.6695
39.5074
39.3357
X : parts per Million : 13C

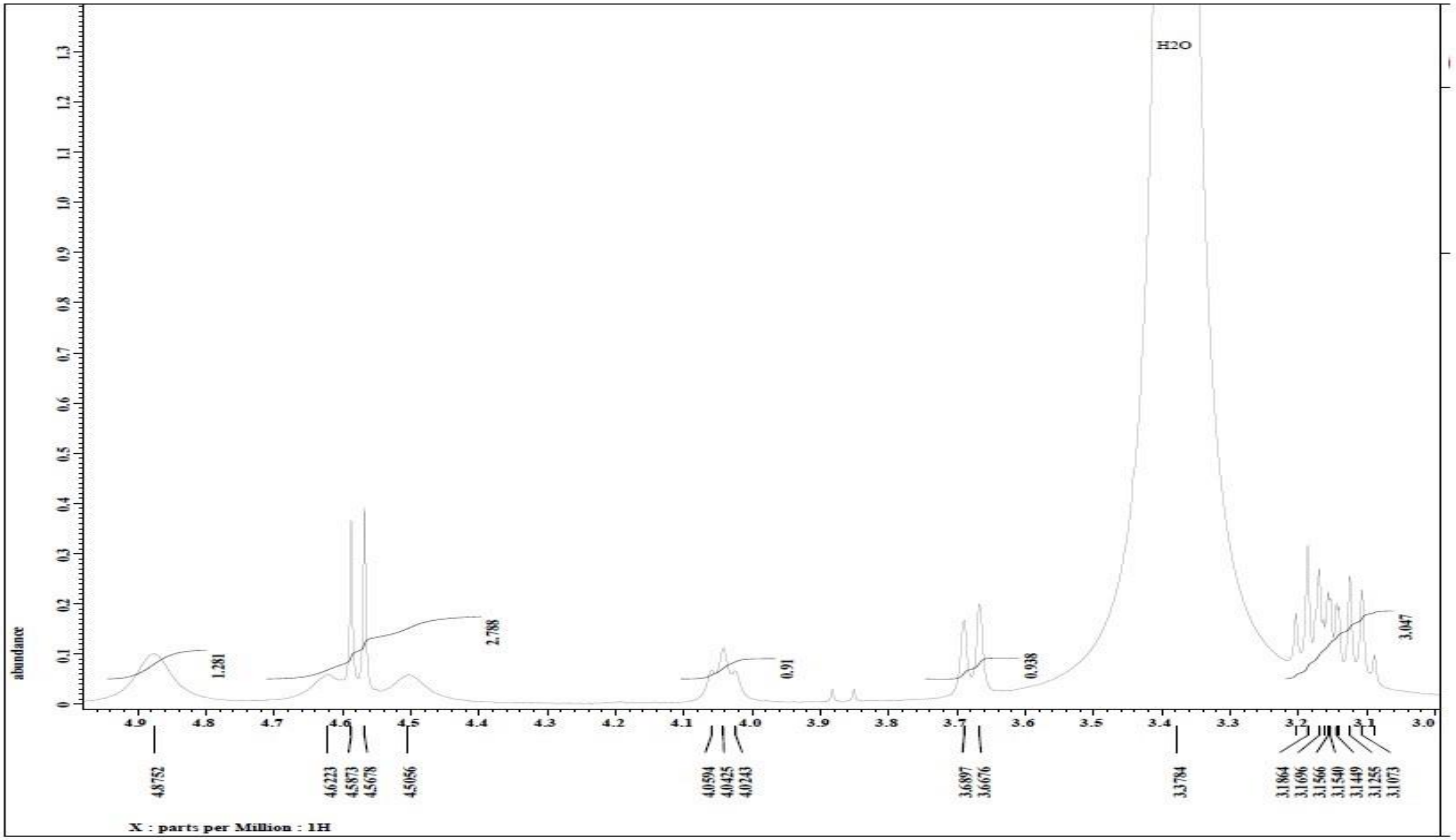

H2O
abundance
1.281
2.788
0.91
0.938
3.047
4.8752
4.6223
4.5873
4.5678
4.5056
4.0594
4.0425
4.0243
3.6897
3.6676
3.3784
3.1864
3.1696
3.1566
3.1540
3.1449
3.1255
3.1073
X : parts per Million : 1H

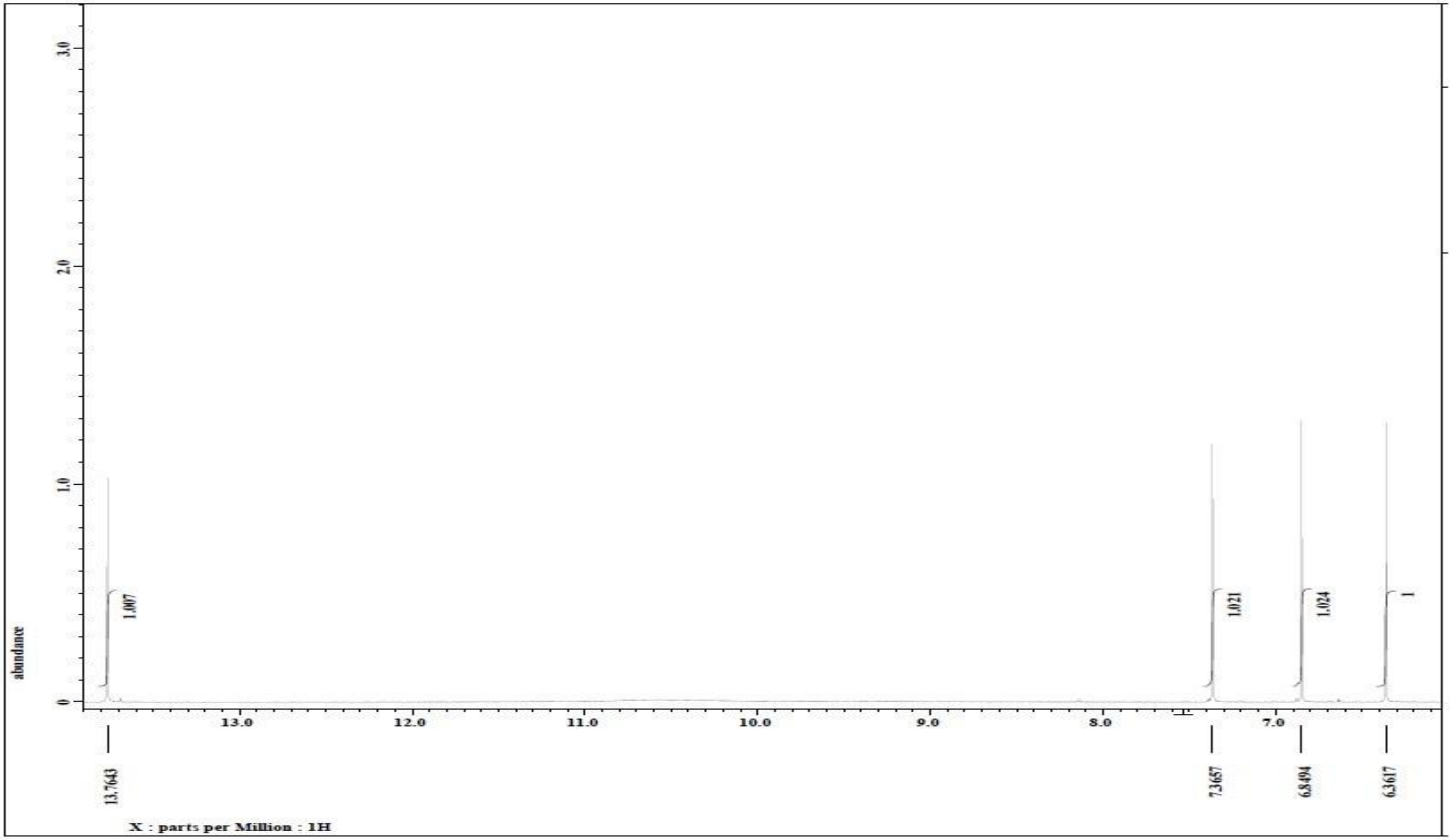

3.0
2.0
1.0
0
abundance
1.007
1.021
1.024
1
13.0
12.0
11.0
10.0
9.0
8.0
7.0
13.7643
7.3657
6.8494
6.3617
X : parts per Million : 1H

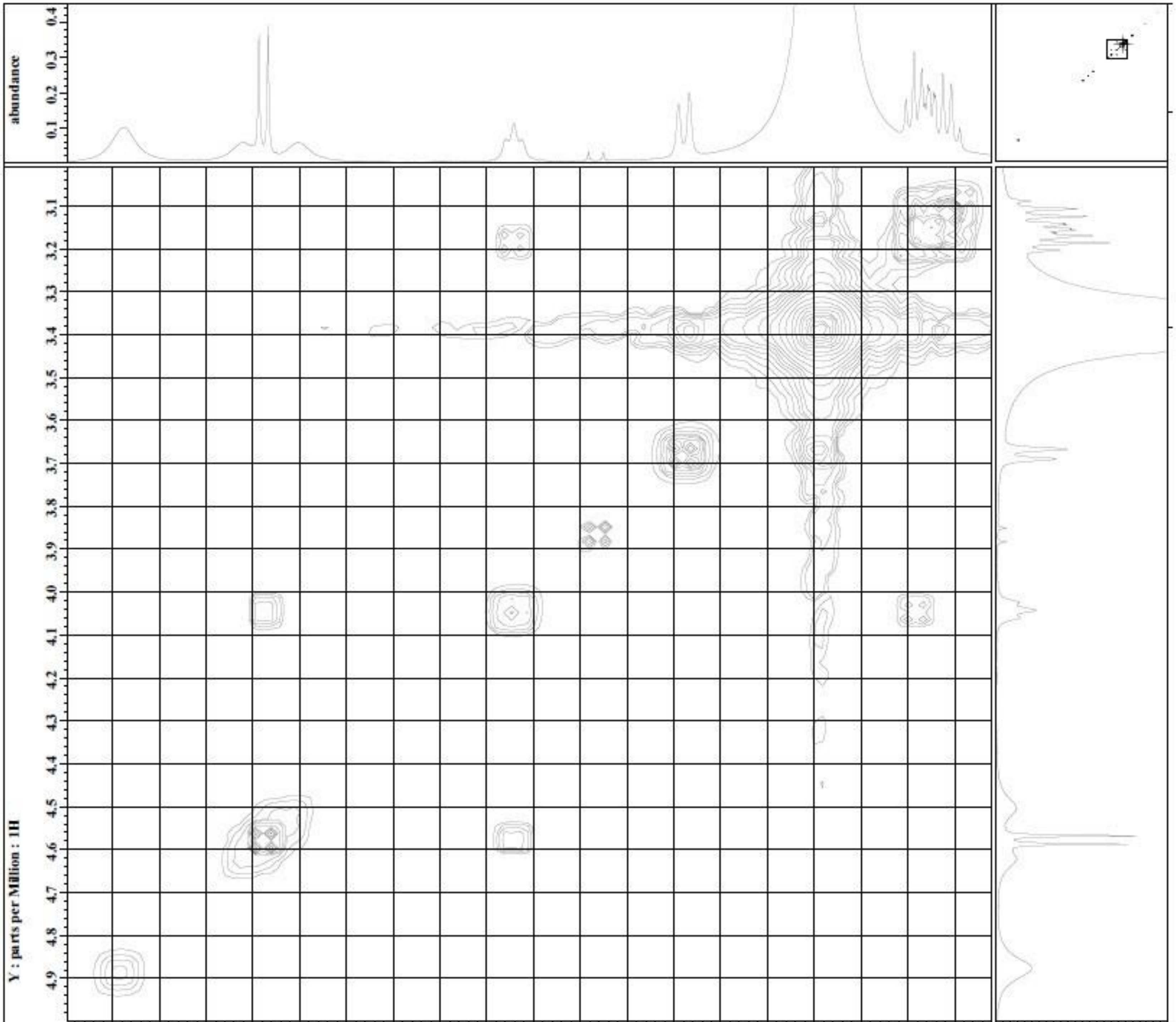

abundance
0.1 0.2 0.3 0.4
Y : parts per Million : 1H
3.1 3.2 3.3 3.4 3.5 3.6 3.7 3.8 3.9 4.0 4.1 4.2 4.3 4.4 4.5 4.6 4.7 4.8 4.9

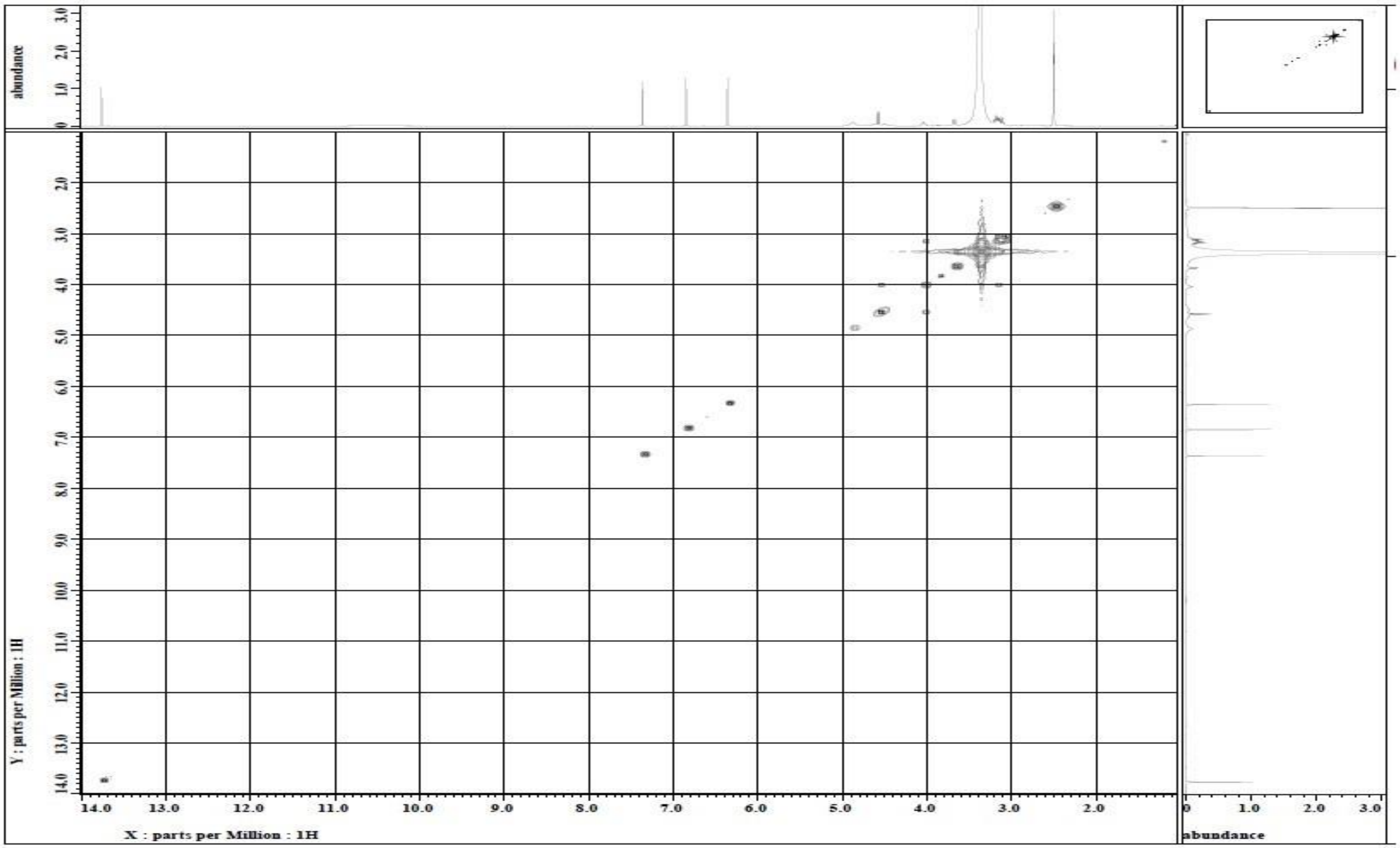

abundance
Y : parts per Million : 1H
X : parts per Million : 1H
abundance

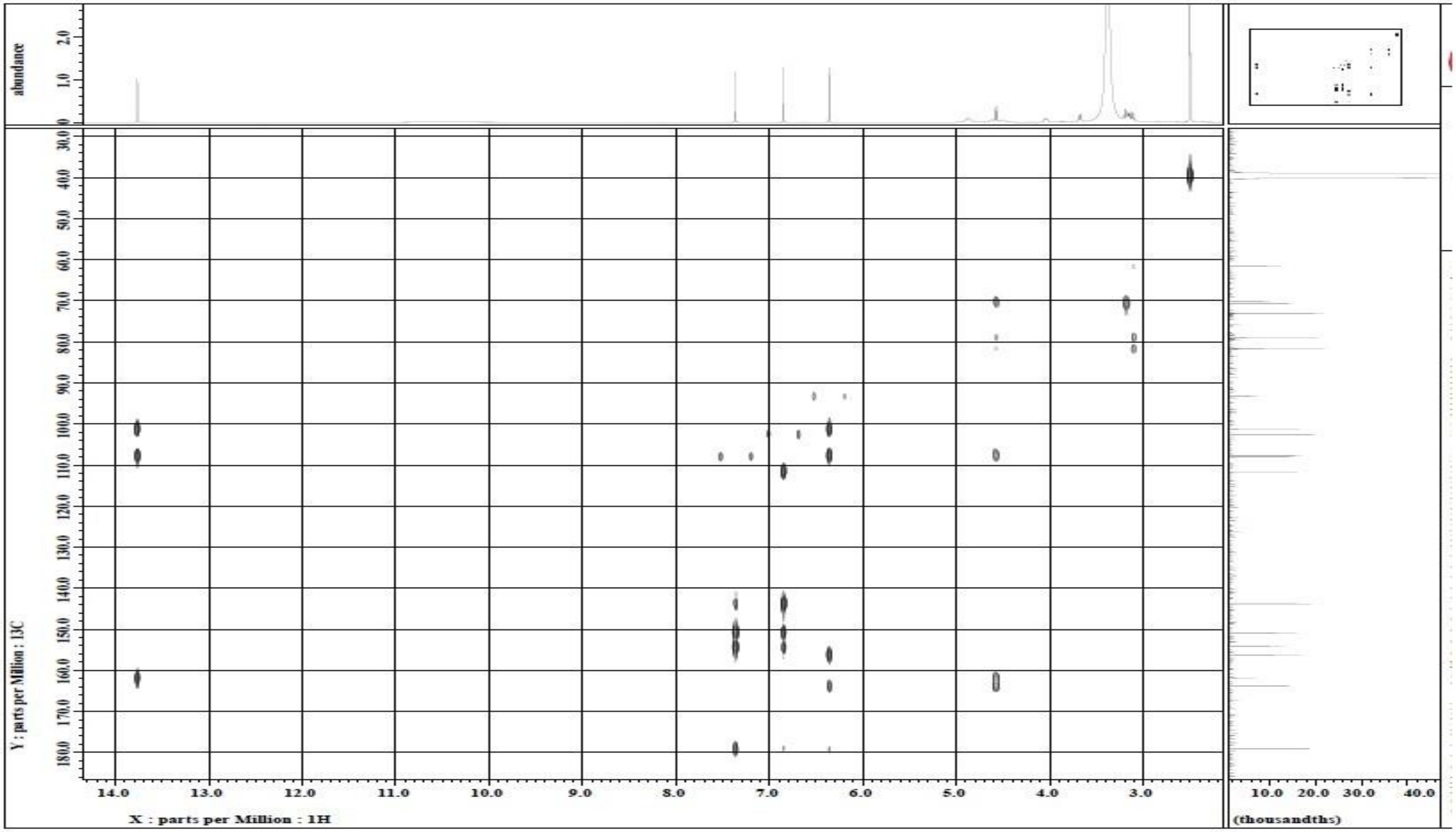
abundance
Y : parts per Million : 13C
X : parts per Million : 1H
(thousandths)

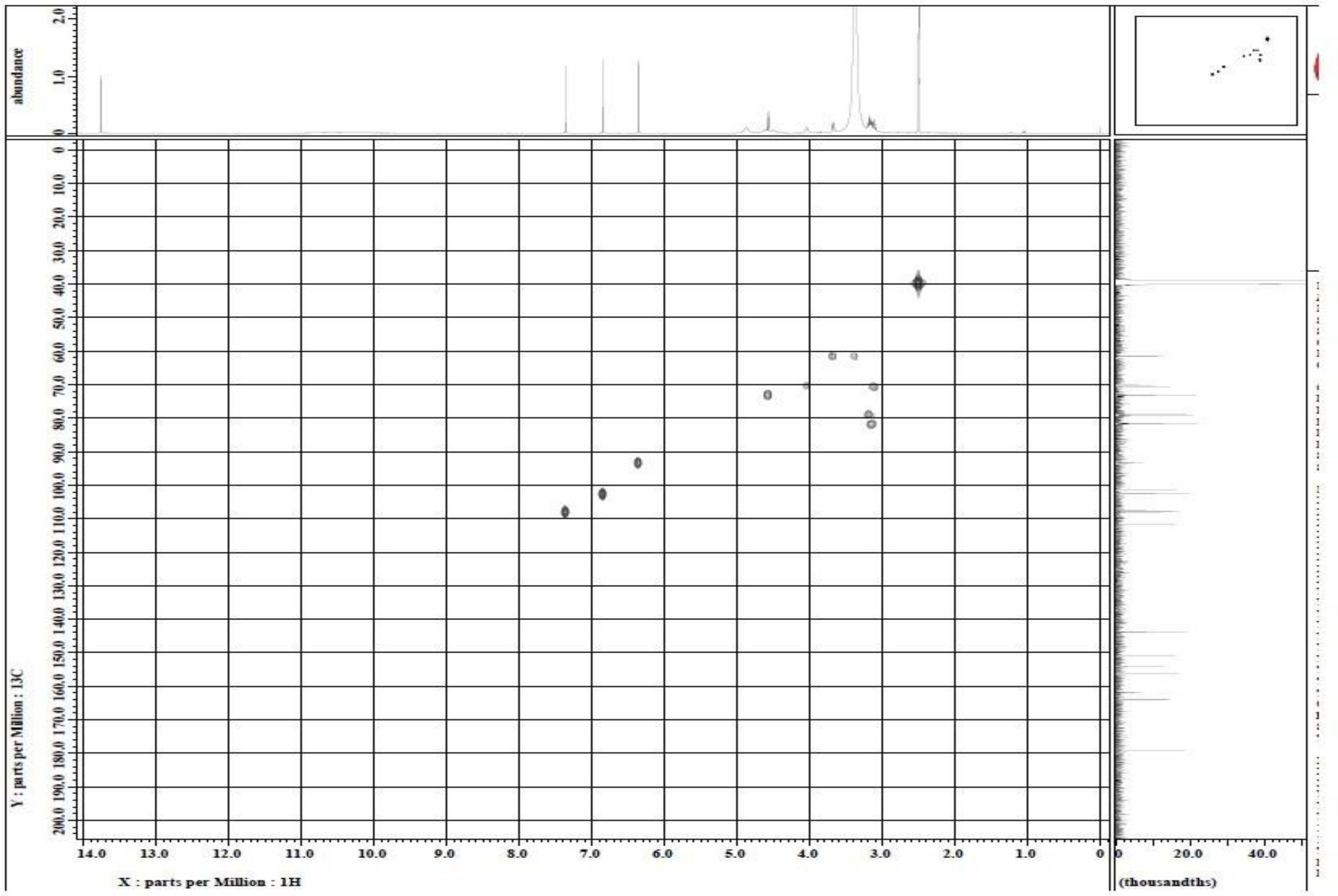
abundance
Y : parts per Million : 13C
X : parts per Million : 1H
(thousandths)
